# Genomes and integrative genomic insights into the genetic architecture of main agronomic traits in the edible cherries

**DOI:** 10.1093/hr/uhae269

**Published:** 2024-09-24

**Authors:** Zhenshan Liu, Anthony Bernard, Yan Wang, Elisabeth Dirlewanger, Xiaorong Wang

**Affiliations:** College of Horticulture, Sichuan Agricultural University, Chengdu 611130, China; INRAE, Univ. Bordeaux, UMR BFP, Villenave d’Ornon 33882, France; College of Horticulture, Sichuan Agricultural University, Chengdu 611130, China; Key Laboratory of Agricultural Bioinformatics, Ministry of Education, Chengdu 611130, China; INRAE, Univ. Bordeaux, UMR BFP, Villenave d’Ornon 33882, France; College of Horticulture, Sichuan Agricultural University, Chengdu 611130, China; Key Laboratory of Agricultural Bioinformatics, Ministry of Education, Chengdu 611130, China

## Abstract

Cherries are one of the economically important fruit crops in the Rosaceae family, *Prunus* genus. As the first fruits of the spring season in the northern hemisphere, their attractive appearance, intensely desirable tastes, high nutrients content, and consumer-friendly size captivate consumers worldwide. In the past 30 years, although cherry geneticists and breeders have greatly progressed in understanding the genetic and molecular basis underlying fruit quality, adaptation to climate change, and biotic and abiotic stress resistance, the utilization of cherry genomic data in genetics and molecular breeding has remained limited to date. Here, we thoroughly investigated recent discoveries in constructing genetic linkage maps, identifying quantitative trait loci (QTLs), genome-wide association studies (GWAS), and validating functional genes of edible cherries based on available *de novo* genomes and genome resequencing data of edible cherries. We further comprehensively demonstrated the genetic architecture of the main agronomic traits of edible cherries by methodically integrating QTLs, GWAS loci, and functional genes into the identical reference genome with improved annotations. These collective endeavors will offer new perspectives on the availability of sequence data and the construction of an interspecific pangenome of edible cherries, ultimately guiding cherry breeding strategies and genetic improvement programs, and facilitating the exploration of similar traits and breeding innovations across *Prunus* species.

## Introduction

Cherry trees are one of the most representative economically important members of the family Rosaceae genus *Prunus*, which contains many famous stone fruits like almonds, apricots, peaches, plums, etc. [1]. Cherry trees include various species valued for three major purposes: as edible fruits for fresh eating or processing like sweet cherry (*Prunus avium* L., syn. *Cerasus avium* (L.) Moench, 2n = 2χ = 16), Chinese cherry (*Prunus pseudocerasus* Lindl., syn. *Cerasus pseudocerasus* (Lindl.) G.Don, 2n = 4χ = 32), sour/tart cherry (*Prunus cerasus* L., syn. *Chlorella vulgaris* Mill., 2n = 4χ = 32), and Nanking cherry (*Prunus tomentosa* Thunb., syn. *Cerasus tomentosa* (Thunb.) Wall., 2n = 2χ = 16), as rootstocks like *Prunus mahaleb* L. (syn. *Cerasus mahaleb* (L.) Mill.), and as blooming ornamentals like *Prunus serrulata* Lindl. (syn. *Cerasus serrulata* (Lindl.) G.Don ex London) [2–4]. In 2022, cherries were grown on 454 664 hectares with a total production of 2 765 827 tons worldwide, with Turkey and China being the largest producer and importer, respectively (www.fao.org/faostat/en/).

There is a large phenotypic variability and genetic diversity among these edible cherries. Sweet cherry is characterized by high fruit weight, large edible proportion, high sugar content, and low acidity, making it suitable for fresh eating [5]. Sour cherry is rich in acids and is valued for superior characteristics in processed products, such as juice, compote, jam, and pie filling [6]. Both sweet and sour cherries are believed to have originated in Europe around the Caspian and Black Seas, with the Caucasus area considered a major center of genetic diversity [7, 8]. Chinese cherry, native to China with wide natural distributions in South China, is characterized by full-flavored but small fruits [9, 10], and contains a diverse range of flavonols, especially kaempferol glycosides and quercetin glycosides [2, 11]. Nanking cherry, native to China, exhibits great adaptability to various environments, with fruits characterized by high total phenolic content (especially flavonols and tannins) and high antioxidant activity [2, 12].

In the last 30 years, breeders worldwide have been working on edible cherries improvement, focusing on fruit quality determining marketability, phenological phases better adapted to climate change, and other high-impact stakeholder-driven production traits such as disease resistance [3, 13–15]. For sweet cherry, hundreds of cultivars are available for growers, with the number of commercial cultivars increasing notably in recent decades [3]. Moreover, a large diversity of cherry landraces are conserved in Genetic Resources Centers (GRCs), such as Cherry Germplasm Repository (Chengdu, China), which mainly conserves Chinese cherry resources, and *Prunus* GRC of INRAE at Bourran (Lot and Garonne, France), which mainly conserves sweet cherry resources, and many have been used for production at a local level, or more recently in modern breeding programs [16].

Breeding has been widely leveraged in many crops for yielding hybrid heterosis [17, 18]. However, high heterozygosity and polygenic control of economically important traits make the dissection of genetic architecture difficult [19], posing a bigger challenge, exacerbated in perennial woody plants and polyploids such as Chinese cherry and sour cherry. Over the past half-century, the release of high-quality genomes and basic molecular technology has advanced our understanding of important aspects of cherry genetics. This includes the consolidation of the phylogenetic relationships, the analysis of the genetic variability, the development of high-throughput genotyping methods, the construction of genetic linkage maps, and the discovery of major genes and quantitative trait loci (QTLs). The Genome Database for Rosaceae (GDR, https: /www.rosaceae.org) [20] made all these data publicly available.

Nevertheless, the use of cherry genomic data is still limited to date, although it could help in understanding the genetic and molecular basis underlying fruit quality, adaptation to climate change, and disease/pest resistance. In this review, we [21] track recent cherry genome achievements and single-nucleotide polymorphism (SNP) arrays, and [19] investigate the trait heredity in genome-wide association studies (GWAS), construction of genetic linkage maps, the discovery of QTLs, and validation of functional genes, and then systematically integrate QTLs, GWAS loci, and functional genes into an identical reference genome to comprehensively demonstrate the genetic architecture of the main agronomic traits. These collective endeavors offer new perspectives on the biology and trait inheritance of edible cherries, aiding in the discovery of genomic loci that control crucial agronomic traits and the construction of an interspecific pangenome of edible cherries, also facilitating the exploration of similar traits and breeding innovations across *Prunus* species.

## Genomic data with SNP arrays of edible cherries

### The *de novo* genomes

The basic chromosome (Chr) number for edible cherries is consistent with that of other *Prunus* species, standing at 8. To date, *P. avium*, *P. cerasus*, and *P. pseudocerasus* have been sequenced (Table 1). The first draft genome sequence of the sweet cherry cv. ‘Satonishiki’ was released in 2017 [22]. It was assembled based on the Illumina platform and showed low heterogeneity. A total of 272.36 Mb in scaffolds were assembled, with an N50 of 0.22 Mb and the longest scaffold of 1.46 Mb, representing 77.8% of the estimated genome size (352.8 Mb). In 2020, three sweet cherry cultivars were sequenced and assembled: cv. ‘Tieton’ (with two versions of the genome [23, 24], cv. ‘Regina’ [25] and cv. ‘Big Star’ [26]. The second version of the sweet cherry cv. ‘Tieton’ greatly improved quality and completeness by integrating multiple technologies: Oxford Nanopore technology (ONT), short Illumina sequencing, and Hi-C scaffolding. The final *de novo* assembly resulted in a phased haplotype assembly of 344.29 Mb with a contig N50 of 3247.2 kb [23]. Recently, an updated complete genome of chromosome-doubled sweet cherry cv. ‘Tieton’ was released [27]. Advanced sequencing technology third-generation circular consensus sequencing (CCS) and Hi-C were applied to assemble a high-quality genome with a total size of 341.62 Mb and an N50 length of 39.81 Mb.

**Table 1:** Summary of edible cherries genomes assembly.

**Species/cultivar: genome version**	**Estimated genome size (Mb)**	**Assembled genome size (Mb)**	**Contig N50 (Mb)**	**Scaffold N50 (Mb)**	**Longest scaffold (Mb)**	**BUSCO (%)**	**# of genes**	**Sequencing platform**	**Reference**
***P. avium* (2n = 2χ = 16)**									
‘Satonishiki’	352.9	272.36	28.779	0.22	1.46	96	43 673	Illumina (HiSeq2000)	[22]
‘Tieton’ V1.0	341.38	280.33	0.064	2.48	17.96	95.9	30 975	Illumina (HiSeq X Ten)	[24]
‘Tieton’ V2.0	340.05	344.29	3.247		/(LG1: 62.32)	97.4	40 338	Illumina (HiSeq X Ten) + Nanopore + Hi-C	[23]
‘Tieton’ T2T		341.62	39.81		/(LG1: 62.94)	98.4	58 204	CCS + HiC	[27]
‘Regina’	338	279	1.230	~6.00	16.3	95.9	39 180	PacBio	[25]
‘Big Star’	322	272.05	0.215	0.19	1.38	95.6	29 487	Illumina (HiSeq2000)	[26]
***P. cerasus* (2n = 4χ = 32, AA’BB)**									
‘Montmorency’	621 (haploid)	1066 (subgenomes A, A’,B)	11.560		/(LG1A: 52.66)	98.6	92 783	Illumina (HiSeq4000) + Pacbio + Nanopore + Hi-C	[30]
***P. pseudocerasus* (2n = 4χ = 32)**									
‘Zhuji Duanbing’ V1.0	1090	359.26	1.11	33.00		97.6	41 811	Illumina (HiSeq X Ten) + Pacbio + Nanopore + Hi-C	[31]
‘Zhuji Duanbing’ V2.0	1090	994.21 (Hap1: 246.30, Hap2: 237.02; Hap3: 225.52; Hap4: 192.91)	7.05 (Hap1: 7.84, Hap2: 6.85; Hap3: 6.00; Hap4: 5.43)	27.26		98.6 (Hap1: 96.00, Hap2: 93.70; Hap3: 88.60; Hap4: 74.30)	Hap1: 26326, Hap2: 25289; Hap3: 24213; Hap4: 20256		

Note: BUSCO: Benchmarking Universal Single-Copy Orthologs were used to assess the completeness of the assembly.

**Table 2:** Genetic linkage maps of edible cherries.

**Origin/mapping parents**	**Pop size**	**Pop type**	**Number of markers**	**Marker type**	**Map size (cM)**	**Linkage groups**	**Mean marker interval (cM)**	**References**
***P. avium***								
‘EF’	56	Microspore-derived callus	89	RAPD	503.3	10	5.6	[46]
‘Regina’ × ‘Lapins’	122	F_1_		SSR		8		[94]
‘NY’ × ‘EF’	86;	F_1_	144	SSR; CAPS; AFLP; SRAP; gene-derived marker	711.1	8	4.9	[95]
‘EF’ × ‘NY’	103		91		565.8	8	6.2	
‘Rainier’ × ‘8–100’	90	F_1_	50	RAPD; ISSR; SSR	634.67	8	12.7	[96]
‘NY54’ × ‘EF’; ‘Regina’ × ‘Lapins’; ‘Namati’ × ‘Summit’; ‘Namati’ × ‘Krupnoplodnaya’	424	F_1_	149	SNP; SSR, Indel, S-Rnase	779.4	8	5.4	[97]
‘Black Tartarian’ × ‘Kordia’	89	F_1_	723	SNP	752.9	8	1.1	[47]
‘Regina’ × ‘Lapins’	121	F_1_	335 (R); 247 (L); 687 (RxL)	SNP	619.4 (R), 610.1 (L), 639.9 (RxL)	8	1.85 (R); 2.47 (L) 0.93 (RxL)	
‘Regina’ × ‘Lapins’	121	F_1_	136 (R); 127 (L)	SNP	712.4 (R); 710.4 (L)	8	5.2 (R); 5.6 (L)	[43]
‘Regina’ × ‘Garnet’	117	F_1_	142 (R); 137 (G)	SNP	657.6 (R); 823.6 (G)	8	4.6 (R); 6.0 (G)	
‘Rainier’ × ‘Rivedel’	675	F_1_	985	SNP; SSR	731.3	8	0.7	[98]
‘Wanhongzhu’ × ‘Lapins’	100	F_1_	718	SNP; SSR; S gene	849	8	1.18	[90]
‘Beniyutaka’ × ‘Benikirari’; ‘C-195-50’ × ‘Benikirari’; ‘Nanyo’ × ‘Benisayaka’	562	F_1_	2382	SNP; SSR	1165	8	0.49	[22]
‘Beniyutaka’ × ‘Benikirari’	94	F_1_	782 (BY); 742 (BK)	SNP; SSR	600.7 (BY); 825.8 (BK)	8	0.77 (BY); 1.11 (BK)	
‘C-195-50’ × ‘Benikirari’	84	F_1_	419 (C-195-50); 625 (BK)	SNP; SSR	745.2 (C-); 926.6 (BK)	8	1.78 (C-); 1.48 (BK)	
‘Nanyo’ × ‘Benisayaka’	304	F_1_	607 (N); 657 (BY)	SNP; SSR	969.3 (N); 1223.5 (BY)	8	1.60 (N); 1.86 (BY)	
‘Vic’ × ‘C’	161	F_1_	816	SNP	726	8	0.9	[41]
‘C’ × ‘C’	97	F_2_	511	SNP	634.1	8	1.7	
‘Brooks’ × ‘C’	67	F_2_	552	SNP	622.4	8	1.2	
‘Fercer’ × ‘X’	67	F_1_	110 (F); 87 (X)	SNP	715 (F); 652.5 (X)	8	6.5 (F); 7.5 (X)	[73]
‘Ambrunés’ × ‘Sweetheart’	140	F_1_	463 (A); 254 (S); 820 (AxS)	SNP	867.8 (A); 529.1 (S); 827.6 (AxS)	8	2.1 (A); 2.4 (S); 1.0 (AxS)	[60]
‘Vic’ × ‘C’; ‘Ambrunés’ × ‘C’; ‘Brooks’(‘B’) × ‘C’; ‘Lambert’ × ‘C’; ‘C’ × ‘C’; ‘B’ × ‘C’-F_2_	406	F_1_; F_2_ (‘C’ × ‘C’; ‘B’ × ‘C’)	1269	SNP	721.98	8	0.57	[77]
‘Vic’ × ‘C’	161	F_1_	910 (Vic); 789 (C); 2000 (VicxC)	SNP	636.7 (Vic); 666.0 (C); 794.3 (VicxC)	8	0.70 (Vic); 0.84(C); 0.37 (VicxC)	[40]
‘Regina’ × ‘Garnet’	454	F_1_	598 (R); 446 (G)	SNP	614.5 (R); 619 (G)	8	1.1 (R); 1.7 (G)	[39]
	1.386 (fine mapping)	F_1_	17	KASP	0.887 kb (9.269–10.156 kb)^*^	1 (LG4)	0.052 kb^*^	

(Continued)

**Table 2:** Continued

**Origin/mapping parents**	**Pop size**	**Pop type**	**Number of markers**	**Marker type**	**Map size (cM)**	**Linkage groups**	**Mean marker interval (cM)**	**References**
***P. cerasus***								
‘RS’ × ‘EB’	86	F_1_	126 (RS); 95 (EB)	SDRF	461.6 (RS); 279.2 (EB)	19 (RS);16 (EB)	3.66 (RS); 2.94 (EB)	[99]
‘RS’ × ‘EB’	86	F_1_	67 (RS); 43 (EB); 80 (RSxEB)	RFLP	398.2 (RS); 222.2 (EB); 272.9 (RSxEB)	15 (RS); 8 (EB); 17 (RSxEB)	9.8 (RS&EB); 4.8 (RS/EB)	[100]
‘M172’ × ‘25–02-29’; ‘Montmorency’ × ‘25–02-29’; ‘25–14-20’ × ‘25–02-29’; ‘ÚjfehértóiFürtös’ × ‘Surefire’; ‘Rheinische Schattenmorelle’ × ‘Englaise Timpurii’	330	F_1_	2058	SNP	659.5	8	0.3	[48]
***P. avium* × *Prunus incisa***								
‘Napoleon’ × ‘E621’	63	F_1_	14	Isoenzyme		4		[101]
***P. avium* × *Psychomyia nipponica***								
‘Napoleon’ × ‘F1292’	47	F_1_	14	Isoenzyme		4		[101]
‘Napoleon’ × ‘F1292’	94	F_1_	166	SSR; gene-specific marker	680	8	3.9	[102]

Note: Pop: population; ‘EF’: ‘Emperor Francis’; ‘RS’: ‘Rheinische Schattenmorelle’; EB: ‘Erdi Botermo’; NY: ‘New York’; ‘C’: ‘Cristobalina’; RAPD: random amplified polymorphic DNA; SDRF: single-dose restriction fragment; RFLP: restriction fragment length polymorphism; SSR: simple sequence repeat; CAPS: cleaved amplified polymorphism sequence; AFLP: amplified fragment length polymorphism; SRAP: sequence-related amplified polymorphism; SNP: single-nucleotide polymorphism; S-gene: self-incompatibility gene. ^*^ The ‘Map size (cM)’ and ‘Mean marker interval (cM)’ of the 17 KASP markers are given in kilobase pairs (kb) of the physical position on the ‘Regina’ genome.

Several studies have demonstrated that sour cherry is a natural hybrid derived from a tetraploid resembling *Prunus fruticosa* (ground cherry) and a diploid resembling *P. avium* [28, 29]. The allotetraploid origin of sour cherry is well established and further supported by the recently published genome: Sour cherry cv. ‘Montmorency’ is trigenomic, containing two distinct subgenomes inherited from ground cherry (A and A’) and two copies of the same subgenome from sweet cherry (BB) [30]. The genome composition of ‘Montmorency’ is AA’BB and little-to-no recombination has occurred between the progenitor subgenomes (A/A’ and B). A total of 92 783 protein-coding genes were predicted in the full assembly of ‘Montmorency’ (1066 Mb) with a high Benchmarking Universal Single-Copy Orthologs (BUSCO) completion score of 98.6% [30].

A chromosome-level, haplotype-resolved genome of Chinese cherry cv. ‘Zhuji Duanbing’ was assembled into 32 pseudochromosomes, 4 haplotypes, comprising 993.69 Mb [31]. Intra-haplotype comparative analyses revealed extensive intra-genomic sequence and expression consistency, confirming that *P. pseudocerasus* was a stable autotetraploid species [4, 32].

### Resequencing efforts and SNP arrays

With the advancement of Next-Generation Sequencing, there has been a thriving progression in the development of SNP arrays [33, 34] and ongoing resequencing studies [35–38]. The first 6 K array, led by RosBREED, consisted of 4214 sweet cherry SNPs and 1482 sour cherry SNPs. However, only a third of them were polymorphic in the sweet and sour cherry evaluation panels, with low coverage in many genomic regions. Subsequently, an additional 9 K SNPs were added onto the 6 K array following a focal point strategy, including SNPs from *fruticosa* subgenomes of sour cherry, respectively [34]. The final 15 K SNPs increased genetic resolution and genome coverage, contributing to a better understanding of the genetic control of key traits. The use of RosBREED Cherry 6 + 9 K SNP array allowed the saturation of previously generated SNP maps in the species, revealing a powerful tool for QTL and genetic analysis [40, 39]. As expected, the number of heterozygous SNPs detected by the 15 K array [40] was larger (1.5–1.9 times) than previously observed for the same cultivars (‘Vic’ and ‘Cristobalina’) with the 6 K array [41]. In essence, these two arrays lie in a foundational work that has significantly advanced modern molecular breeding research of cherries [43, 42–45]. The first whole-genome resequencing was conducted to characterize genetic variation, population structure, and allelic variants in a panel of 21 sweet cherries, which encompassed the majority of cultivated Greek germplasm and a representative of a local wild cherry elite phenotype [38]. These expanded sequencing datasets, combined with those genomes, greatly increase the value of cherry breeding and advance our understanding of their evolution, diversity, and the genetic control of valuable traits.

## Genetics and functional genomics of edible cherries

### Development of genetic linkage maps

Genetic linkage maps are composed of molecular markers, the order of which is arranged based on genetic distance estimated from recombination events between individuals. They serve as a powerful tool for exploring and understanding the inheritance of agronomic traits from parents. The history and development of genetic maps for edible cherries are summarized in Table 2, representing the concerted efforts of geneticists and breeders worldwide. Most of the genetic maps are based on the analysis of F_1_ populations derived primarily from crosses between sweet cherry accessions. The first genetic map was constructed on sweet cherry using random amplified polymorphic DNA (RAPD) and isoenzyme marker analysis of 56 microspore-derived callus individuals of the cv. ‘Emperor Francis’ [46]. Prior to the development of the first 6 K SNP array, studies focused on developing and densifying frameworks using long-fragment markers such as simple sequence repeat (SSR), cleaved amplified polymorphism sequence (CAPS), amplified fragment length polymorphism (AFLP), sequence-related amplified polymorphism (SRAP), RAPD, restriction fragment length polymorphism (RFLP), etc. It was not until Klagges *et al.* [47] used two F_1_ intra-specific populations of sweet cherry, ‘Black Tartarian’ × ‘Kordia’ (BT × K) and ‘Regina’ × ‘Lapins’ (R × L), that two SNP-based highly dense maps consisting of 723 and 687 markers were constructed, containing eight linkage groups spanning 752.9 and 639.9 cM with an average distance of 1.1 and 0.9 cM of BT × K and R × L, respectively.

Currently, >20 bi-parental populations of sweet cherries are used for genetic mapping, including F_1_ and a minimal number of F_2_ populations. The population sizes range from 47 to 1386 individuals, with the largest population (1386) used for fine mapping [39]. The consensus genetic map, released concurrently with the first sweet cherry genome, holds the record for the highest number of markers (2382) in the sweet cherry genetic maps [22]. In contrast to the extensive mapping efforts in sweet cherry, genetic maps for non-sweet cherry species have generally featured a low number of individuals and genetic markers except Cai *et al.* [48], who developed a high-density consensus map for sour cherry by integrating independent maps from over five mapping populations, a total of 2058 markers covering 659.5 cM, which represents a mean marker interval of 0.3 cM.

Comparative analysis of this genetic map confirms, for the first time, a high level of chromosome synteny between sweet cherry and peach, providing evidence for a reciprocal application of genetic knowledge between peach and cherry. Most of these genetic maps and genetic markers have been collected and integrated into GDR. The high-quality and high-density linkage maps are still expected to be constructed for edible cherries, which could enhance the mapped QTL number as well as the precision.

### QTL identification for varied traits

In the past 25 years, significant progress has been made in QTL mapping studies for various traits in edible cherries, with GDR already collecting >300 QTLs. Here, we review 94 highly reliable QTLs from sweet and sour cherries, as no QTLs have been reported on other edible cherries. The selection criteria include: (i) twice the natural log of Bayes factors (2lnBF) >5 or logarithm of odds ratio (LOD) >3, (ii) percentage of variance explained (*PVE*) >5%, and (iii) detection in at least 2 years. With the exception of two studies [40, 49] reporting QTLs from a single year, Calle *et al.* [40] stand out as the only study focusing on precise phenotyping of chemical compounds related to fruit color by using high-performance liquid chromatography (HPLC) in edible cherries, which posed significant challenges in data collection. Despite the limitation of 1-year data, Rosyara *et al.* [49] identified powerful QTLs crucial for fruit size, including the well-known *FW_G2a* locus. The haplotype combinations of SSR markers BPPCT034 and CPSCT038, both located within the *FW_G2a* interval, have been confirmed as effective indicators for determining fruit size at the early stages [50].

Based on inputs from the cherry community, QTLs for 30 quantitative traits have been identified so far (Table 3). These traits were classified into four main categories: biochemical (including seven compounds related to anthocyanin biosynthesis), fruit quality (encompassing traits related to fruit color, size, firmness, cracking, soluble solid content, and acidity), phenology (covering chilling requirement, flowering date, maturity date, and fruit development period), and physiology (focused on trunk diameter). The abbreviations and categories were proposed by referring to terms in the Plant Trait Ontology in GDR, with some modifications. In particular, we counted the number of years in which reliable QTLs were detected in single-year analysis (SY) or multi-year analysis (MY). Very stable QTLs across 10 years were reported for the flowering date (FD) [39].

**Table 3:** List of highly reliable QTLs in edible cherries (only reported in sweet cherry and sour cherry).

**Category/sub-category (trait)**	**QTL label** ^**1**^	**Label in GDR** ^**2**^	**Mapping population**	**Linkage group**	**QTL interval (cM)**	**QTL peak (cM)**	**LOD/2lnBF** ^**3**^	**PVE** ^**3**^	**Nb of years in SY/MY analysis** ^**3**^	**Reference**
**Biochemical**										
Cyanidin 3-O-glucoside	q1_CY3G	qCYG.VC6 + 9–3.2	‘Vic’ × ‘Cristobalina’	C3	41.9–59.45		3.1	11.9	SY: 1	[40]
	q2_CY3G	qCYG.VC6 + 9–3.1		V3	27.22–32.84		3.5	13.7	SY: 1	
Cyanidin 3-O-rutinoside	q3_CY3R	qCYR.VC6 + 9–3.2		C3	35.95–53.82		4.8	18.9	SY: 1	
	q4_CY3R	qCYR.VC6 + 9–3.1		V3	20.39–31.56		5.5	22.7	SY: 1	
Neochlorogenic acid	q5_Neochlorogenic acid	qNA.VC6 + 9–1.1		V1	141.34–142.9		19.9	60.3	SY: 1	
	q6_Neochlorogenic acid	qNA.VC6 + 9–3.1		V3	8.24–19.38		4	6.8	SY: 1	
p-Coumaric acid	q7_p-CA	qCA.VC6 + 9–1.1		V1	141.34–141.63		23.6	67.9	SY: 1	
p-Coumaroyl quinic acid	q8_p-CQA	qCQA.VC6 + 9–1.1		V1	141.34–142.9		32.5	77.9	SY: 1	
Peonidin 3-O-glucoside	q9_Pe3G	qPEG.VC6 + 9–3.1		V3	19.39–31.53		3.6	16.4	SY: 1	
Peonidin 3-O-rutinoside	q10_Pe3R	qPER.VC6 + 9–3.2		C3	45.17–48.39		5.3	21.8	SY: 1	
	q11_Pe3R	qPER.VC6 + 9–4.1		C4	54.04–61.58		4.1	18.4	SY: 1	
**Fruit quality**										
Fruit color (flesh)	q12_FC (flesh)	qFFC.VC6 + 9–3.2-2019	’Vic’ × ‘Cristobalina’	C3	32.47–82.33		12.3	32.2	SY: 1	[40]
	q13_FC (flesh)	qFFC.VC6 + 9–3.1-2019		V3	17.64–48		12.4	32.2	SY: 1	
	q14_FC (flesh)		‘New York 54’ × ‘Emperor Francis’	LG3		55.4	45.74	94.7	SY: 3	[103]
Fruit color (skin)	q15_FC (skin)	qFSC.VC6 + 9–3.2-2018	‘Vic’ × ‘Cristobalina’	C3	27.77–70.54		12.3	31.9	SY: 3	[40]
	q16_FC (skin)	qFSC.VC6 + 9–3.1-2018		V3	20.38–47.00		12.8	34.9	SY: 3	
	q17_FC (skin)	qFSC.EN-ch3.2	‘New York 54’ × ‘Emperor Francis’	LG3		54.2	27.8	86.8	SY: 3	[103]
	q18_FC (skin)	qFSC.EN-ch3.2		LG3		55.4	28	87.1	SY: 3	
Fruit cracking	q19_FCr	qFRCRK-LG1.1 m-multi	Multifamily	LG1	44–55	45		10.5	MY: 2/SY: 2	[51]
	q20_FCr	qFRCRK-LG5.1 m-multi		LG5	44–53	48		8.1	MY: 2/SY: 2	
Fruit cracking (fruit side)	q21_FCr (FS)		‘Regina’ × ‘Garnet’	R2	2.1–8.9	5.5	37	15.2	MY: 7/SY: 2	[45]
	q22_FCr (FS)			R2	11.8–76.7	47.6	37	15.2	MY: 7/SY: 2	
	q23_FCr (FS)		‘Regina’ × ‘Lapins’	L7	0–7.2	2.4	28.3	11.6	MY: 7/SY: 2	
	q24_FCr (FS)			L7	72.7–77.8	76.5	28.3	11.6	MY: 7/SY: 2	
Fruit cracking (pistillar end)	q25_FCr (PE)		‘Regina’ × ‘Garnet’	G4	15.3–26.3	20.8	29	13.7	MY: 7/SY: 2	
	q26_FCr (PE)			G4	79.5–95.2	93.9	29	13.7	MY: 7/SY: 2	
	q27_FCr (PE)		‘Regina’ × ‘Lapins’	R5	1.4–6.7	4	79.9	17.4	MY: 7/SY: 3	

(Continued)

**Table 3:** Continued

**Category/sub-category (trait)**	**QTL label** ^**1**^	**Label in GDR** ^**2**^	**Mapping population**	**Linkage group**	**QTL interval (cM)**	**QTL peak (cM)**	**LOD/2lnBF** ^**3**^	**PVE** ^**3**^	**Nb of years in SY/MY analysis** ^**3**^	**Reference**
q28_FCr (PE)		‘Regina’ × ‘Garnet’	R5	11.5–18.1	14.8	66.9	17.3	MY: 7/SY: 3		
q29_FCr (PE)		‘Regina’ × ‘Lapins’	R5	34.7–39.4	37	79.9	17.4	MY: 7/SY: 3		
q30_FCr (PE)		‘Regina’ × ‘Garnet’	R5	43.5–54	48.8	66.9	17.3	MY: 7/SY: 3		
Fruit cracking (stem end)	q31_FCr (SE)		‘Fercer’ × ‘X’	X6	53.5–77.7	65.6	21.4	18.6	MY: 7/SY: 2	
q32_FCr (SE)		X6	77.2–90.5	83.8	21.4	18.6	MY: 7/SY: 2			
Fruit firmness	q33_FF	qFRFRM-LG1.2 m-multi	Multifamily	LG1	34–70	48		21.8	MY: 3/SY: 3	[51]
q34_FF	qFRFRM.A-ch1.1-Y1	‘Ambrunés’ × ‘Sweetheart’	A1	60.30–76.29		4.08	18.8	SY: 2	[60]	
q35_FF	qFRFRM.S-ch1.2-Y1	S1	16.84–30.76		5	22.5	SY: 2			
q36_FF	qFRFRM-LG3.2 m-multi	Multifamily	LG3	53–66	62		9.1	MY: 3/SY: 3	[51]	
q37_FF	qFRFRM-ch4.1-multi	Multifamily	LG4	50–54		11.7/9.5*	47.9/64.1	SY: 2	[42]	
q38_FF	qFRFRM.FX-X4.1	‘Fercer’ × ‘X’	X4	33.1–36.0	34.5	125.3	70.2	MY: 7/SY: 7	[73]	
q39_FF	qFRFRM.FX-F4.1	F4	10.3–67.7	39	20.6	20.1	MY: 7/SY: 7			
q40_FF		‘Regina’ × ‘Garnet’	R5	13.9–67.7	44.7	25.5	24.1	MY: 4/SY: 4	[58]	
q41_FF	-	R5	67.7–67.7	67.7	25.5	24.1	MY: 4/SY: 4			
Fruit size	q42_FS	qFRSZ-ch2.1-multi	multifamily	LG2	57–76		6.7/7.2*	23.6/21.5	SY: 2	[42]
Fruit size (fruit longitude diameter)	q43_FS (FLoD)		‘New York 54’ × ‘Emperor Francis’	EF2		28.9	6	26	SY: 2	[59]
q44_FS (FLoD)		NY2		41.4	7.7	19.4	SY: 2			
q45_FS (FLoD)		NY6		56.8	11.2	37.8	SY: 2			
Fruit size (fruit transverse diameter)	q46_FS (FTrD)		EF2		30.9	7.4	24.5	SY: 2		
q47_FS (FTrD)		NY2		44.4	7.3	26.1	SY: 2			
q48_FS (FTrD)		NY6		56.8	7.7	39.4	SY: 2			
Fruit size (fruit weight)	q49_FS (FW)	FRW.A-ch1.1-Y2	‘Ambrunés’ × ‘Sweetheart’	A1	101.76–129.84		3.87	17.4	SY: 2	[60]
q50_FS (FW)	qFRW.full-sib_families-LG1.	Four full-sib families	LG1		41	5.7*		SY: 1	[49]	
q51_FS (FW)	qFRW.full-sib_families-LG2.1	LG2		12.9	33.4*		SY: 1			
q52_FS (FW)	qFRW.full-sib_families-LG2.2	LG2		20.9	33.4*		SY: 1			
q53_FS (FW)	qFRW.full-sib_families-LG2.3	LG2		37	5.4*		SY: 1			

(Continued)

**Table 3:** Continued

**Category/sub-category (trait)**	**QTL label** ^**1**^	**Label in GDR** ^**2**^	**Mapping population**	**Linkage group**	**QTL interval (cM)**	**QTL peak (cM)**	**LOD/2lnBF** ^**3**^	**PVE** ^**3**^	**Nb of years in SY/MY analysis** ^**3**^	**Reference**
q54_FS (FW)		‘Regina’ × ‘Lapins’	R2	3.7–22.9	13.3	54	18.3	MY: 7/SY: 7	[58]	
q55_FS (FW)		R2	18–61.2	39.6	54	18.3	MY: 7/SY: 7			
q56_FS (FW)		‘New York 54’ × ‘Emperor Francis’	EF2		21.4	10.8	54	SY: 3	[59]	
q57_FS (FW)		NY2		48.9	14.3	44.7	SY: 3			
q58_FS (FW)	qFRW.full-sib_families-LG3	Four full-sib families	LG3		69	11.9*		SY: 1	[49]	
q59_FS (FW)	qFRW.full-sib_families-LG6	LG6		57	31.6*		SY: 1			
q60_FS (FW)		‘New York 54’ × ‘Emperor Francis’	NY6		56.8	7.9	32.4	SY: 3	[59]	
Fruit size (mesocarp cell number)	q61_FS (MCN)		EF2		19.9	5.1	31.9	SY: 2		
Fruit size (mesocarp transverse diameter)	q62_FS (MTrD)		EF2		28.9	4.9	21.2	SY: 2		
q63_FS (MTrD)		NY2		42.4	6	34.6	SY: 2			
Fruit size (mesocarp longitude diameter)	q64_FS (MLoD)		EF2		30.9	6.6	24.5	SY: 2		
q65_FS (MLoD)		NY2		38.4	9.9	58.1	SY: 2			
Fruit size (pit transverse diameter)	q66_FS (PTrD)		NY6		57.4	6.4	39.4	SY: 2		
Fruit size (pit longitude diameter)	q67_FS (PLoD)		NY6		58.7	9	35.8	SY: 2		
Soluble solid content	q68_SSC	qSSC-ch4.1-multi	Multifamily	LG4	50–59		11.7/6.8*	34.2/22.1	SY: 2	[42]
Total acidity	q69_TA	qTA-ch6.1-multi	LG6	91–108		9.6/6.3*	21.6/15.0	SY: 2		
**Phenology**										
Chilling requirement	q70_CR	qCR.RG-R4. multi-year	‘Regina’ × ‘Garnet’	R4	18.9–23.1	21	20.8	17.5	MY: 3/SY: 3	[43]
Flowering date	q71_FD		‘Regina’ × ‘Garnet’ pop2	G1	49.13–50.88	155.5	20.0	6.1	MY: 3/SY: 3	[39]
**q72_FD** ^**a**^	qBD.M172x25-LG1.2	**M172 × 25–02-29; ‘Montmorency’ × 25–02-29**	LG1	77.8–93.1	85.9	3.5		MY: 3	[48]	
q73_FD		‘Regina’ × ‘Garnet’ pop1	G1	101.7–151.2	128.7	24.1	7.3	MY: 10/SY: 4	[39]	
q74_FD		‘Regina’ × ‘Lapins’	L1b	40–47	46.1	27	20.6	MY: 4/SY: 4	[78]	
q75_FD	qBT-ch1.1-BLUP	Multi-family	LG1	137–139	137	average: 9.5*	32.4	MY: 4/SY: 4	[77]	
**q76_FD** ^**a**^	qBD.US-LG2.1	**‘Újfehértói Fürtös’ ×** ‘**Surefire’**	LG2	20.9–43.0	33	3.2		MY: 3	[48]	
q77_FD	qBT-ch2.1-BLUP	Multi-family	LG2	73–75	75	average: 18.8*	15.2	MY: 4/SY: 4	[77]	

(Continued)

**Table 3:** Continued

**Category/sub-category (trait)**	**QTL label** ^**1**^	**Label in GDR** ^**2**^	**Mapping population**	**Linkage group**	**QTL interval (cM)**	**QTL peak (cM)**	**LOD/2lnBF** ^**3**^	**PVE** ^**3**^	**Nb of years in SY/MY analysis** ^**3**^	**Reference**
q78_FD	qFD.RG-R4. multiyear	‘Regina’ × ‘Garnet’	R4	18.4–22.5	20.4	76.1	36.3	MY: 5/SY: 5	[43]	
q79_FD		‘Regina’ × ‘Garnet’ pop1	R4	19–22.2	20.6	146.1	34.3	MY: 10/ SY: 10	[39]	
**q80_FD** ^**a**^	qBD.US-LG4.1	**‘Újfehértói Fürtös’ × ‘Surefire’**	LG4	26.8–34.1	33.7	3.2		MY: 3	[48]	
q81_FD	qFD.RL-R4.miltiyear	‘Regina’ × ‘Lapins’	R4	27.9–31.2	29.6	47	21.2	MY: 6/SY: 6	[43]	
q82_FD		‘Regina’ × ‘Lapins’	R4	33–34	33.2	57.2	47.2	MY: 4/SY: 4	[78]	
**q83_FD** ^**a**^	qBD.M172x25-LG5.1	**M172 × 25–02-29**	LG5	11.6–34.4	25.7	3.5		MY: 3	[48]	
q84_FD		‘Regina’ × ‘Garnet’ pop1	R7	34.7–57.6	54.6	31.9	5.7	MY: 10/ SY: 7	[39]	
Flowering date (beginning)	q85_FD (beginning)		‘Regina’ × ‘Lapins’	L1	136.9–152.2	145.9	39.2	7.2	MY: 3–5 (ML: 5)	[76]
q86_FD (beginning)		R4	0–0.5	29.4	149.6	20.9	MY: 3–5 (ML: 5)			
q87_FD (beginning)		L6	8.6–23.4	16	42.2	7.3	MY: 3–5 (ML: 5)			
Fruit development period	q88_FDP	qFDT-ch4.2-multi	Multi-family	LG4	51–53		10.8/11.7*	65.3/64.5	SY: 2	[42]
Maturity date		q89_MD	qDMAT-ch2.1-multi	LG2	68–76		6.1/11.7*	11.7/10.4	SY: 2	
q90_MD	qDMAT-ch4.2-multi	LG4	51–53		9.5/11.8*	46.8/52.5	SY: 2			
**Physiology**										
Trunk diameter	q91_TD	qTRDIA.WL-LG7.2012.2	‘Wanhongzhu’ × ‘Lapins’	LG7		80.4	3.61	21.1	SY:2	[90]
q92_TD	qTRDIA.WL-LG7.2012.3	LG7		80.8	3.29	17.3	SY: 2			
q93_TD	qTRDIA.WL-LG7.2012	LG7		78	3.24	15.7	SY: 2			
q94_TD	qTRDIA.WL-LG8.2012	LG7		41.5	4.28	21.5	SY: 3			

Note: ^1^ QTL label with ^a^ is detected from sour cherry, others are detected from sweet cherry; ^2^ the names of QTLs included in the GDR; ^3^ highly reliable QTLs are with strong evidence (the two times the natural log of Bayes factors (2lnBF) >5 or logarithm of odds ratio (LOD) >3 and percentage of variance explained (PVE) >5) over at least 2 years except for two studies ([40]; [49]) with QTLs from only 1 year. Calle *et al.* [40] is the sole study localizing chemical compounds in edible cherries, posing data collection challenges. Rosyara *et al.* [49] provide crucial insights into cherry genetics despite 1-year data; ^3^ the number of years/sites in which QTL was detected in single-year analysis (SY) or multi-year analysis (MY) in multi-sites (MS); * represent the data come from 2lnBF; −: data unavailable;.

### Marker-trait association identified from GWAS

GWAS were reported on three sweet cherry germplasm collections covering >600 accessions: 116 from INRAE *Prunus* Genetic Resources Center at Bourran (Lot and Garonne, France) [35], 235 from the Research and Breeding Institute of Pomology Holovousy Ltd [36], and 259 from Washington State University [51]. We reviewed 118 major GWAS loci from 17 traits with strong evidence: *P-*value <.05 and *PVE* > 3 (Table S1). They are classified into fruit quality, phenology, and physiology categories. Donkpegan *et al.* [35] used three reference genomes, one peach genome ‘PLov2-2n’ V2.0 and two sweet cherry genomes ‘Regina’ and ‘Satonishiki’, to conduct SNP–trait associations, Crump *et al.* [51] used *Prunus persica* var. ‘PLov2-2n’ V2.0, and Holušová *et al.* [36] used *P. avium* var. ‘Tieton’ V2.0. Regarding GWAS models, the performance of fixed and random model circulating probability unification (FarmCPU) was highlighted in datasets from Donkpegan *et al.* [35] and Holušová *et al.* [*36*]. Bayesian-information and linkage-disequilibrium iteratively nested keyway (Blink) and multi-locus mixed model (MLMM) were also employed by Crump *et al.* [51] and Donkpegan *et al.* [35], respectively. The use of multi-genome and multi-model strategy certainly strengthens the validity of the identified associations, and the consistent identification of major loci across these different models and genomes suggests a degree of convergence and reliability in the findings.

### Genes conferring validated function

In general, the related traits display continuous phenotypic variation and are governed by multiple quantitative trait genes (QTGs), which can be identified by map-based cloning or GWAS [52]. Numerous candidate genes have been identified in the edible cherries’ QTL intervals and upstream/downstream regions of the peak-associated loci. However, none of the QTGs have been functionally validated, and reverse genetic research is preferred to validate diverse biological and physiological functions of genes identified through comparative transcriptomic analysis. Also, they could serve as molecular keys to elucidate differences in traits, thus offering insights for genetic studies. Moreover, they could be explored for applications in molecular-assisted breeding (MAB) and molecular-assisted selection (MAS), and provide foundational information underlying molecular mechanisms.

As of June 2024, we have collected 48 genes cloned from edible cherries, including two genes from sour cherry (*PcSOT1* and *PcSOT2*), four from Chinese cherry (*CpARF7, CpCHS1, PpsGalAK-like*, and *PpsStv1*), and 42 from sweet cherry (Table 4). These genes were classified into five categories and 15 sub-categories based on their involvement in a specific trait: abiotic stress (5 genes), biochemical (3 genes), fruit quality (24 genes), phenology (11 genes), and physiology (5 genes). The gene names are consistent with the nomenclature in the reference.

**Table 4:** List of experimentally validated genes in edible cherries.

**Category/sub-category**	**Gene** ^**1**^	**Chr**	**Position (Tieton V2.0) (Mb)**	**Function validated**	**Gene (protein) description**	**References**
**Abiotic stress**						
Abiotic stress	*CpARF7^b^*	7	26.34	Regulate drought and low phosphorus stress and root formation	Auxin response factor 7	[104]
Drought tolerance	*CpCHS1^b^*	1	4.66	Enhance drought resistance	Chalcone synthase	[105]
	*PacCYP707A1*	5	17.69	Mediate drought tolerance	Abscisic acid 8′-hydroxylase 1-like	[85]
Drought & salt tolerance	*PaLectinL16*	4	14.96	Enhance resistance with abiotic (salt, drought) stresses	Lectin receptor-like kinases	[92]
Salt tolerance	*PaLectinL7*	2	40.05	enhance salt tolerance; promoted lignin deposition	lectin receptor-like kinases	[106]
**Biochemical**						
Fruit sorbitol and dry matter	*PcSOT1^a^*	8	25.16	Regulate sorbitol and dry matter accumulation	Sorbitol transporter	[107]
	*PcSOT2^a^*	8	25.18	Regulate sorbitol and dry matter accumulation	Sorbitol transporter	
Cherry allergy	*PacLTP*	6	33.34	Elicit cherry allergy (for Mediterranean population)	Non-specific lipid-transfer protein	[108]
**Fruit quality**						
Fruit color	*PavGST1*	3	5.11	Promote fruits’ anthocyanin accumulation	Glutathione S-transferase F11-like	[70]
	*PacMYBA*	3	24.00	Promote anthocyanin accumulation in red-colored fruit	R2R3 MYB transcriptional regulator	[68]
	*PavMYB10.1*	3	24.00	Determine fruit colors	R2R3-MYB transcription factor MYB10.1 protein	[66]
	*PavBBX6*	3	30.30	Promote anthocyanin accumulation	B-box zinc finger protein 21-like	[53]
	*PavNCED1*	4	12.22	Promotes anthocyanin biosynthesis; promote ABA biosynthesis	9-cis-epoxycarotenoid dioxygenase NCED1	([68]; [54])
	*PavBBX9*	4	12.71	Promote anthocyanin accumulation	B-box zinc finger protein 22	[53]
	*PacCOP1*	5	19.95	Negatively regulate anthocyanin biosynthesis	E3 ubiquitin-protein ligase COP1-like	[69]
Fruit cracking	*PaPIP1–4*	2	40.52	Prevent cracking by pre-harvest calcium treatments	Probable aquaporin PIP1–4	[75]
Fruit firmness (and rippen)	*PpsGalAK-like^b^*	1	9.16	Increase protopectin content	Galacturonic acid-1-phosphate kinase	[31]
	*PavDof2*	1	44.39	Delay softening	Dof zinc finger protein DOF3.4	[54]
	*PavDof6*	2	44.31	Precocious, promote softening	Dof zinc finger protein DOF4.6-like	
	*PaMADS7*	3	32.12	Positively regulate fruit ripening and softening	MADS-box transcription factor	([54]; [74])
	*PavARF8*	4	3.43	Delayed softening	Auxin response factor 3	[54]
	*PavPG38*	4	8.59	Reduce fruit firmness	Polygalacturonase	[71]
	*PavNAC56*	4	16.01	Promote ripening and softening	NAC transcription factor 25	[70]
	*PavDof15*	5	33.18	Delayed softening	Dof zinc finger protein DOF5.4	[54]
	*PavXTH14*	6	10.78	Reduce fruit firmness	Xyloglucan endotransglucosylase/hydrolase	[71]
	*PpsStv1^b^*	6	13.13	Increase protopectin content	Homologous of glycosyltransferases 29 family	[31]
	*PavXTH15*	8	33.07	Reduce fruit firmness	Xyloglucan endotransglucosylase/hydrolase	[71]
Fruit size	*PavAGL15*	2	9.12	Increase fruit size	Agamous-like MADS-box protein AGL15	[62]
	*PaCYP78A9*	2	40.11	Increase fruit size	Cytochrome P450 78A9-like	[63]
	*PavRAV2*	3	31.27	Decrease fruit size (mesocarp cell expansion)	AP2/ERF and B3 domain-containing transcription repressor RAV2-like	[61]
	*PaCYP78A6*	5	28.23	Increase fruit size	Cytochrome P450 78A9-like	[64]
	*PavKLUH*	5	33.03	Increase fruit size (mesocarp cell expansion)	Pentatricopeptide repeat-containing protein PNM1	[61]

(Continued)

**Table 4:** Continued

**Category/sub-category**	**Gene** ^**1**^	**Chr**	**Position (Tieton V2.0) (Mb)**	**Function validated**	**Gene (protein) description**	**References**
**Phenology**						
Flowering date (dormancy)	*PavGA2ox-2 L*	1	13.69	Delays flowering time, promote dwarf dense planting and inhibits seed germination	Gibberellin 2-beta-dioxygenase 2-like	[81]
	*PavSEP*	3	32.12	Shorten vegetative phase and promote early flowering	Developmental protein SEPALLATA 1	[82]
	*PavNCED5*	4	5.42	Enhanced seed and flower bud dormancy	Probable 9-cis-epoxycarotenoid dioxygenase NCED5	[84]
	*PavCIG1*	5	25.25	Delayed flowering	Dehydration-responsive element-binding protein 1E-like	[87]
	*PavCIG2*	5	25.26	Repress flowering and maintain the dormancy status	Dehydration-responsive element-binding protein 1E	
	*PavFUL*	5	33.10	Led to early flowering and multi-silique formation	Truncated transcription factor CAULIFLOWER A	[83]
	*PavTCP17*	5	33.53	Positively regulate flower bud dormancy	Hypothetical protein	[86]
	*PavSVP*	6	26.16	Maintain suppression phase of flowering	MADS-box protein SVP	[82]
	*PavFT*	6	37.56	promote flowering	protein HEADING DATE 3A	[109]
Maturity date	*PacCYP707A2*	7	22.51	Negatively regulate cherry fruit ripening	Abscisic acid 8′-hydroxylase 4-like	[85]
**Physiology**						
Flower and seed	*PavDAM1*	1	54.64	Result in abnormal flower and seed development	MADS-box protein JOINTLESS-like	[80]
	*PavDAM5*	1	54.70	Result in abnormal flower and seed development	MADS-box protein JOINTLESS-like	
	*PavSOC1*	2	33.89	Result in abnormal flower and seed development	MADS-box protein SOC1	[80]
Plant hormone signaling	*PaNRT2.1*	6	10.53	Regulate nitrate signaling pathways	High-affinity nitrate transporter 2.1-like	[91]
	*PaLAX1*	6	31.72	Promote cell uptake of auxin	Auxin transporter-like protein 2	[110]
Self-incompatibility	*S4-SLFL2*	6	34.99	Mediate the ubiquitination and degradation of S-RNase	S locus F-box protein	[89]

Note: Genes with multiple function are categorized into the first appearing category based on the order of function validated; ^1^ genes with ^a^ are cloned from sour cherry, genes with ^b^ are validated in Chinese cherry, others are cloned from sweet cherry.

It is well known that the difficulty in functionally validating genes is a significant challenge in genetic transformation of stone fruit trees. However, *PavBBX6* and *PavBBX9* were successfully overexpressed in sweet cherry calli to enhance light-induced anthocyanin biosynthesis and abscisic acid (ABA) accumulation [53]. Many genes, like *PavDof2/6/15*, *PavNAC56*, *PpsGalAK-like*, and *PpsStv1* were successfully transiently overexpressed in sweet cherry fruit [31, 54].

## Genetic architecture of traits by integrating QTLs, GWAS loci, and functional genes

**Figure 1:**
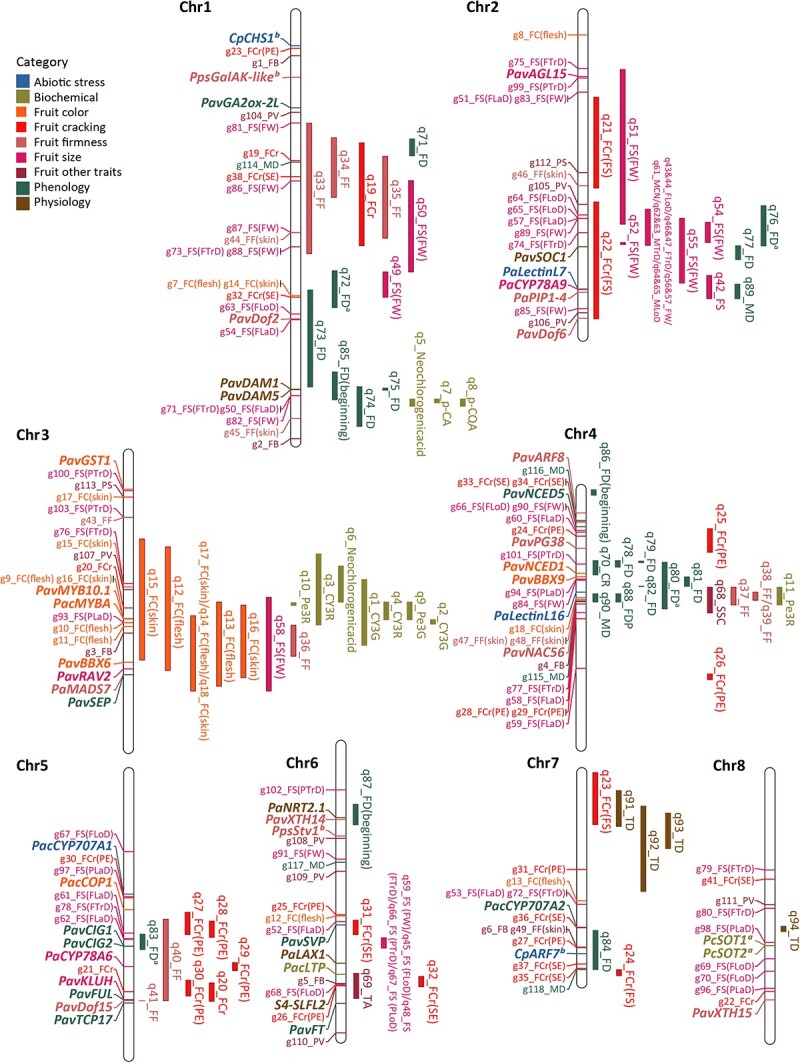
Genetic architecture of traits by integrating QTLs (Table 3), GWAS loci (Table S1) and functional genes (Table 4) into the reference genome ‘Tieton’ V2.0. On the right side of the chromosome, each QTL delimits an approximate physical range based on LOD support intervals of QTL identified from different studies. GWAS loci and functional genes are present to the left of the chromosome, and the functional genes are in bold italics. Detailed information is available in Table S2.

Genetic architecture refers to the characteristics of genetic variation that drive heritable phenotypic variability. It is influenced by the number of genetic variants affecting a trait, population frequencies, the magnitude of their effects, and their interactions with each other and the environment [55]. Understanding the genetic architecture of complex traits is a central goal of cherry genetics research. Here, we drew a comprehensive genetic architecture detailing the traits of edible cherries by integrating QTLs, GWAS loci, and functional genes into the reference genome *P. avium* ‘Tieton’ V2.0 (Fig. 1, Table S2).

The highly reliable QTLs originate from genetic maps with varying resolutions as well as GWAS data spanning across different genomes. This fragmentation of genetic information across various sources hampers our ability to comprehensively analyze and understand complex trait architectures. To overcome this challenge, we conducted a genome-wide alignment to the *P. avium* ‘Tieton’ V2.0 genome by using BLAST on GDR (https://www.rosaceae.org/blast/nucleotide/nucleotide) with default parameters. For QTLs, the physical positions were predominantly determined by aligning marker sequences at both ends of the QTL interval. For GWAS loci, sequences within 1000 bp upstream and downstream to the peak SNP were extracted for alignment. TBtools-II [56] was employed for extracting sequences. Validity was assessed based on an identity percentage >90% for all alignment entries; if not met, sequences within 2000 bp upstream and downstream were utilized. For functional genes, coding sequences or primer sequences were used for alignment. This work will lead to enhanced genetic resources, thereby accelerating genetic discovery for cherry improvement.

### Fruit size and fruit weight

The size and weight of cherry fruits are crucial factors determining their overall quality and appeal to consumers, often described using terms like longitude (length), transverse (diameter/width), and lateral diameter (thickness), etc. This dimensionality largely depends on a coordinated series of cell divisions and expansions in the fleshy mesocarp [57]. We reviewed 26 reported QTLs underlying fruit size and weight across chromosomes 1, 2, 3, and 6. Among these 26 QTLs, 17 (*q42*, *q43*, *q44*, *q46*, *q47*, *q51* to *q57*, and *q60* to *q65*) were co-localizing in a narrow interval on Chr2, including peak markers BPPCT034 and CSPSCT038 [49, 58, 59]. Moreover, Holušová *et al.* [36] confirmed five fruit size-related GWAS SNPs (*g57, g64, g65, g74, g89* in Fig. 1 and Table S2) positioned around 29 Mb on Chr2, within the ‘hot’ QTL interval of fruit size, exhibiting strong associations (*p* < 1.0^e−10^). Hence, new QTL or GWAS was discovered near the ‘hot’ interval; it draws the attention of breeders and geneticists. Additionally, two fruit weight QTLs, *q50_FS (FW)* [60] and *q49_FS (FW)* [49], share an overlapping region of ~1.8 Mb on Chr1. Pit size-related QTLs (*q66_FS (PTrD)* and *q67_FS (PLoD)*) on Chr6 were located at the same position as QTLs for fruit diameter (*q45_FS (FLoD)* and *q48_FS (FTrD)*) and fruit size QTL *q60_FS (FW)* ([59]. The strong genetic linkage between fruit size and pit size highlights the potential difficulty in implementing an effective genetic improvement strategy that would involve the increasing of flesh area while simultaneously decreasing the pit size. Notably, five genes were confirmed to regulate sweet cherry fruit size through virus-induced gene silencing. *PavRAV2* [61] and *PavAGL15* [62] are negative regulators, and they suppressed fruit enlargement by weakening mesocarp cell expansion and cell cycling and proliferation, respectively. *PaCYP78A9* is likely to be an important upstream regulator of cell cycle processes and *PaCYP78A6* acts redundantly with *PaCYP78A9* to affect fruit size [63, 64]. Silencing *PavRAV2* resulted in enlarged fruit due to enhanced mesocarp cell expansion. More precisely, the mesocarp cell length and volume in the *PavRAV2*-silenced sweet cherry fruits increased by 58% and 26%, respectively, compared with those of the control fruits [61]. Significantly, we observed that *PavRAV2* is located within the QTL *q58_FS(FW)*, which has a large additive effect (~1.6 g) on fruit size [49]. This suggests that further in-depth investigation of gene *PavRAV2* could be crucial for explaining the genetic variation in fruit size. Besides, silencing *PavKLUH* resulted in decreased fruit size by restricting mesocarp cell expansion [61].

### Fruit color

Edible cherry fruits exhibit a range of rich colors from white to black purple, directly influencing their commercial value. By compiling previously reported results, Chr3 plays a crucial role in controlling cherry fruit color, housing six GWAS loci, seven QTLs, and four validated genes related to flesh or skin color, and fruit coloration in edible cherries results from anthocyanin accumulation [11, 65]. The QTLs for essential metabolites regulating anthocyanin biosynthesis pathway are also located on Chr3 [40] (Fig. 1, Table S2). All fruit color QTLs on LG3 shared a common 6.43-Mb interval (from the top of *q14/17/18* to the bottom of *q15*). As expected, the *R2R3 MYB* transcription factor *PavMYB10.1* is located in this region, which has been confirmed as the major determinant in anthocyanin biosynthesis. Three different alleles, *PavMYB10.1c*, *PavMYB10.1b*, and *PavMYB10.1a*, determine yellow, blush, and dark-red colors, respectively. Color segregation in two F_1_ populations conforms to Mendel first segregation law in a 3:1 ratio and a 1:1 ratio, revealing that *PavMYB10.1a* was dominant to *PavMYB10.1b* [66]. Metabolomic profiling of cherries has demonstrated that cyanidins play a dominant role in fruit color determination [11, 67]. Notably, *PavMYB10.1* is also located in three QTLs for cyanidins (*q3_CY3R*, *q4_CY3R* and *q1_CY3G*) [40]. Therefore, it can be inferred more precisely that *PavMYB10.1* determines cherry color by modulating cyanidin synthesis in the anthocyanin biosynthesis pathway, thus providing insights into refining the regulatory network and genetic mechanisms underlying cherry color.

Another noteworthy location for regulating cherry color is the middle of Chr4, where two validated genes (*PavNCED1* and *PavBBX9*) [68, 53, 54] were identified within the 2 Mb interval upstream of the QTL for peonidin 3-O-rutinoside [40] (*q11_Pe3R*: 14.17–16.76 Mb) (Table S2). *PavNCED1* is an important gene in the ABA biosynthesis pathway, with its expression promoted by *PavBBX6* and *PavBBX9*, which together positively regulate light-induced anthocyanin accumulation [53]. Therefore, peonidin 3-O-rutinoside, capable of imparting a deep red or purple color to the fruit, is speculated to be the specific anthocyanin compound regulated by *PavNCED1* and *PavBBX9*. Several other genes have been confirmed to participate in color formation. *PacCOP1* negatively regulates anthocyanin biosynthesis, while *PavGST1* positively regulates sweet cherry fruit anthocyanin accumulation, with its transcription being activated by *PavMYB10.1* [70, 69].

### Fruit cracking and firmness

Cracking is the most severe abiotic threat to profitability in sweet cherries, and high firmness ensures post-harvest quality and adequate shelf life. Among sweet cherry fruit quality traits, firmness has been rated third in importance by consumers, above size and color (Zheng *et al.*, 2016). These processes are modulated by a set of cell wall-modifying enzymes, the most studied of which include xyloglucan endotransglucosylase/hydrolases (XTHs) and polygalacturonases (PGs). Genetic architecture of fruit cracking and firmness is complex, with QTLs and GWAS loci widely distributed across seven chromosomes except the eighth chromosome. Among 18 *PavXTHs* and *45 PavPGs*, *PavXTH14*, *PavXTH15*, and *PavPG38* significantly reduce the fruit firmness through modification of pectin and hemicellulose [71]. MADS-box genes constitute a highly conserved family of transcription factors and are involved in floral organogenesis, flowering time, embryo and fruit development, and ripening. Qi *et al.*, [72] revealed that *PaMADS7* binds to the promoter of *PaPG1*, serving as an indispensable positive regulator of sweet cherry fruit ripening and softening.

On Chr4, a validated NAC transcription factor (*PavNAC56*) is located in three highly reliable QTLs for firmness (*q37_FF*, *q38_FF*, and *q39_FF*), with *q38_FF* being particularly noteworthy as it was identified in MY and SY analysis for 7 years with maximum LOD of 125 and PVE of 75% [73, 42]. *PavNAC56* plays an indispensable role in controlling the ripening and softening of sweet cherry fruit. It directly binds to the promoters of several genes related to cell wall metabolism (*PavPG2*, *PavEXPA4*, *PavPL18*, and *PavCEL8*) and activates their expression [74]. On Chr 2, *PaPIP1;4*, together with *PavDof6*, is located within the *q22_FCr(FS)* interval. *PaPIP1;4* is the only plasma membrane aquaporin validated to prevent cherry fruit cracking, which is upregulated by pre-harvest calcium treatment [75]. ABA-responsive transcription factor *PavDof6* results in precocious ripening of sweet cherry fruit. Along with *PavDof2/15*, *PavDof6* is activated by auxin response factor *PavARF8*, and they all directly bind to the *PavNCED1* promoter and regulate its expression, forming a feedback mechanism for ABA-mediated cherry fruit softening [54]. *PavDof15*, located on Chr5, has the same function as *Pavdof2* in delaying cherry fruit softening, and it is also located within the interval of firmness and cracking-related QTLs (*q40_FF*, *q20_FCr*, and *q41_FF*). On LG1, there are four QTLs related to firmness and cracking (*q33_FF*, *q34_FF*, *q19_FCr*, *q35_FF*), sharing a notable 5.99-Mb overlap interval. Within this region lies an SNP, *g19_FCr*, which exhibits a high PVE of 12.7% [51], underscoring its considerable potential for further exploration.

### Phenology

Phenology traits and their findings will enable increased efficiency of breeding strategies focused on adaptation to future climatic conditions. Phenology traits like FD and maturity date were reported to be highly polygenic, and many QTLs with high PVE values were identified [44]. The end of Chr1 and the middle of Chr4 are identified as two ‘hotspot’ regions where FD QTLs from both sweet cherry and sour cherry aggregate, according to previous studies [43, 77, 39, 48, 42, 78, 76] (Fig. 1, Table S2). However, within these two ‘hotspot’ QTL intervals, no GWAS loci neither validated genes have been reported yet. Particularly, MADS-box transcription factors have been identified as strong candidate genes for the genetic control of blooming and temperature responses in many species, with structural mutations in *PavDAMs* validated as a DNA-based marker for selection [79]. However, in sweet cherry, functional and expression analyses of *PavDAM1/5* showed that they might have a role in flower development instead of endodormancy induction and bud formation [80].

Nevertheless, there are still 11 functional genes related to phenology distributed on the first seven chromosomes. *PavGA2ox-2 L* functions as a GA metabolic gene that promotes dwarf dense planting, delays flowering time, and inhibits seed germination [81]. The overexpression of *PavSEP* and *PavFUL* in *Arabidopsis* leads to early flowering promotion [82, 83], and *PavNCED5* promoted flower bud dormancy [84]. The negative regulator *PacCYP707A2* and positive regulator *PavTCP17* are both involved in regulating ABA synthesis, but act on bud dormancy and fruit ripening in sweet cherry, respectively [85, 86]. C-repeat binding factor (CBF) plays an important role in response to low temperature. Two novel CBF homologous genes of sweet cherry, *PavCIG1* and *PavCIG2*, were isolated with the function of repressing flowering, falling within the narrow *q83_FD* (~2.2 Mb) interval on Chr5 [48, 87]. *PavFT* is expressed during floral bud determination and can promote flowering in a winter-annual Arabidopsis accession [83]. The MADS-box transcription factor *PavSVP* exhibits the functions in maintaining the suppression phase of flowering [82].

### Physiological and abiotic stress traits

Self-incompatibility (SI) of cherry species is genetically controlled by a single polymorphic S locus, which harbors a single S-RNase as the pistil S determinant and several F-box genes as pollen S determinants ([21]; Matsumoto *et al.*, 2012). Moreover, the insertion of the putative M locus-encoded GST (MGST) promoter region also probably leads to the SC in the cultivar ‘Cristobalina’ despite lacking functional validation [88]. Precisely, *S4-SLFL2* plays a role in mediating the ubiquitination and degradation of S-RNase, leading to SC in the cultivar ‘Lapins’ [89].

Trunk diameter (TD) is a physiological trait correlated with tree vigor, resistance, and fruit yield. Four QTLs on Chr7 and Chr8 provide genetic information for controlling this trait [90]. *PaNRT2.1* mediates dark septate endophyte (DSE)-dependent nitrogen assimilation in sweet cherry roots [91].

Several genes for abiotic stress traits have been validated through reverse genetics, including two cloned from Chinese cherry: *CpCHS1* increases drought tolerance (Hou *et al.*, 2022), and *CpARF7* responds to drought tolerance and low phosphorus (Hou *et al.*, 2022). *PaLectinL16* enhances sweet cherry resistance with salt stress [92], and so on (Table 4). Overall, all these studies demonstrate that any issue affecting cherry growth, development, productivity, and geographic adaption is one that needs to be addressed.

## Concluding remarks and prospects

We summarized the current progress in edible cherries genomes and genomics, focusing on genetic maps, QTLs, GWAS loci, and validated functional genes. By integrating these genetic findings into the ‘Tieton’ V2.0 genome, a global genetic architecture of main agronomic traits was established, which could be served as a reference for further advancements in edible cherries. However, species-specific challenges remain. Current research mainly focused on diploid sweet cherries, particularly on fruit quality and phenological traits. However, enhancing abiotic stress tolerance is a pressing challenge in the context of global climate change. The complex tetraploid genomes of sour cherry and Chinese cherry have slowed genetic progress, yet their recent genome sequences offer opportunities to address limitations like small fruit size, soft texture, and short shelf life. Genetic studies on Nanking cherry (*P. tomentosa*) remain limited, but its unique shrub form and the medicinal properties of its seeds [93] provide promising avenues for future exploration.

Alongside the increasing availability of genomic resources and growing analytical approaches such as multi-omics, it will be possible to pinpoint the genomic regions, the genes, and the molecular mechanisms involved in the genetic determinism of traits of interest in edible cherries. Moreover, the genomic transferability among *Prunus* species leads this review to a possible broader application. Insights obtained about a gene associated with a specific trait in one edible cherry species can be extrapolated to another species within the genus, facilitating the exploration of similar traits across different species. The co-localizations integrated in this review will enable researchers to undertake comparative studies more efficiently, thereby accelerating the transfer of knowledge and breeding innovations across *Prunus* species. Finally, the availability of sequence data and targeted genomic regions involved in the variation of a trait will allow the construction of an interspecific pangenome of edible cherries, and a pangenome will make it possible to identify common genomic regions under selection pressure in common between the species. The ‘hot’ regions for specific traits could ultimately guide cherry breeding strategies and genetic improvement programs.

## Supplementary Material

Web_Material_uhae269

## Data Availability

All the data is presented in the text file.
