# Supplementary material for: Genomes and integrative genomic insights into the genetic architecture of main agronomic traits in the edible cherries
Source: Hortic Res. 2024 Sep 24;12(1):uhae269. doi: 10.1093/hr/uhae269 (PMC11718393; doi:10.1093/hr/uhae269)
Supplement: Web_Material_uhae269 [file web_material_uhae269.zip › Table S1&S2.docx]

**Table S1. List of major loci identified using GWAS in edible cherries (only reported in sweet cherry)**

| **Category/Sub-Category (trait)** | **GWAS label** | **Model** | ***P*-value^1^** | **PVE^1^** | **Origin position** | **Genome/Reference** |
| --- | --- | --- | --- | --- | --- | --- |
| **Fruit quality** | | | | | | |
| Fruit bruiseness | g1_FB | FarmCPU | 3.81E-11 | NA | chr1_6099740 | *P. avium* 'Tieton' V2.0 (Holušová *et al*., 2023) |
|  | g2_FB | FarmCPU | 2.55E-21 | NA | chr1_61827812 |  |
|  | g3_FB | FarmCPU | 2.80E-09 | NA | chr3_26046798 |  |
|  | g4_FB | FarmCPU | 3.06E-37 | NA | chr4_16229770 |  |
|  | g5_FB | FarmCPU | 1.06E-09 | NA | chr6_34665604 |  |
|  | g6_FB | FarmCPU | 4.61E-09 | NA | chr7_25384148 |  |
| Fruit color (flesh) | g7_FC (flesh) | FarmCPU | 1.60E-14 | NA | chr1_41003304 |  |
|  | g8_FC (flesh) | FarmCPU | 2.14E-11 | NA | chr2_3072021 |  |
|  | g9_FC (flesh) | FarmCPU | 3.15E-35 | NA | chr3_23939472 |  |
|  | g10_FC (flesh) | FarmCPU | 3.58E-16 | NA | chr3_24544649 |  |
|  | g11_FC (flesh) | FarmCPU | 3.71E-22 | NA | chr3_25100670 |  |
|  | g12_FC (flesh) | FarmCPU | 6.07E-11 | NA | chr6_24854866 |  |
|  | g13_FC (flesh) | FarmCPU | 3.35E-12 | NA | chr7_18661295 |  |
| Fruit color (skin) | g14_FC (skin) | FarmCPU | 2.25E-11 | NA | chr1_41003304 |  |
|  | g15_FC (skin) | FarmCPU | 1.01E-14 | NA | chr3_19315249 |  |
|  | g16_FC (skin) | FarmCPU | 1.37E-41 | NA | chr3_23939472 |  |
|  | g17_FC (skin) | FarmCPU | 4.76E-09 | NA | chr3_6272221 |  |
|  | g18_FC (skin) | FarmCPU | 5.31E-30 | NA | chr4_15747406 |  |
| Fruit cracking | g19_FCr | Blink | <0.05 | 12.7 | Pp01_16000000 | *P. persica* ‘PLov2-2n’ V2.0 (Crump *et al*., 2022) |
|  | g20_FCr | Blink | <0.05 | 3.6 | Pp03_15900000 |  |
|  | g21_FCr | Blink | <0.05 | 13.8 | Pp05_15500000 |  |
|  | g22_FCr | Blink | <0.05 | 6.6 | Pp08_18100000 |  |
| Fruit cracking (pistillar end) | g23_FCr (PE) | FarmCPU | 1.64e−09 | 11.4 | PAV01_REGINA_759551 | *P. avium* ‘Regina’ (Donkpegan *et al.*, 2023) |
|  | g24_FCr (PE) | MLMM | 1.38e−09 | 8.9 | PAV04_REGINA_6606690 |  |
|  | g25_FCr (PE) | FarmCPU | 6.48e−09 | 7.7 | PAV06_REGINA_22196234 |  |
|  | g26_FCr (PE) | FarmCPU | 1.10e−07 | 3.2 | PAV06_REGINA_31626357 |  |
|  | g27_FCr (PE) | FarmCPU | 6.15e−09 | 6.9 | PAV07_REGINA_24643127 |  |
|  | g28_FCr (PE) | MLMM | 9.98e−15 | 9.0 | Pp04_13790367 | *P. persica* ‘PLov2-2n’ V2.0 (Donkpegan *et al*., 2023) |
|  | g29_FCr (PE) | FarmCPU | 6.80e−16 | 9.0 | Pp04_13790367 |  |
|  | g30_FCr (PE) | FarmCPU | 1.39e−08 | 7.4 | Pp05_1152140 |  |
|  | g31_FCr (PE) | FarmCPU | 7.49e−11 | 4.9 | Pp07_7874904 |  |
| Fruit cracking (stem end) | g32_FCr (SE) | FarmCPU | 2.00e−07 | 20.9 | PAV_r1.0chr1_26464729 | *P. avium* 'Satonishiki' (Donkpegan *et al*., 2023) |
|  | g33_FCr (SE) | MLMM | 1.13e−10 | 30.9 | PAV_r1.0chr4_4381656 |  |
|  | g34_FCr (SE) | FarmCPU | 1.38e−14 | 30.9 | PAV_r1.0chr4_4381656 |  |
|  | g35_FCr (SE) | FarmCPU | 2.98e−07 | 9.6 | PAV_r1.0chr7_17821793 |  |
|  | g36_FCr (SE) | MLMM | 1.13e−10 | 5.9 | PAV07_REGINA_21757494 | *P. avium* ‘Regina’ (Donkpegan et al., 2023) |
|  | g37_FCr (SE) | FarmCPU | 4.88e−07 | 3.9 | PAV07_REGINA_26772180 |  |
|  | g38_FCr (SE) | MLMM | 1.13e−10 | 4.8 | Pp01_18149428 | *P. persica* ‘PLov2-2n’ V2.0 (Donkpegan *et al*., 2023) |
|  | g39_FCr (SE) | FarmCPU | 2.12e−09 | 5.5 | Pp01_29966902 |  |
|  | g40_FCr (SE) | FarmCPU | 4.82e−09 | 6.0 | Pp08_11366089 |  |
|  | g41_FCr (SE) | FarmCPU | 4.13e−07 | 16.0 | Pp08_6129863 |  |
| Fruit firmness | g42_FF | Blink | <0.05 | 19.2 | Pp01_15300000 | *P. persica* ‘PLov2-2n’ V2.0 (Crump *et al*., 2022) |
|  | g43_FF | Blink | <0.05 | 6.0 | Pp03_4500000 |  |
| Fruit firmness (skin) | g44_FF(skin) | FarmCPU | 9.66E-10 | NA | chr1_31855038 | *P. avium* 'Tieton' V2.0 (Holušová *et al*., 2023) |
|  | g45_FF(skin) | FarmCPU | 7.35E-09 | NA | chr1_58902546 |  |
|  | g46_FF(skin) | FarmCPU | 2.63E-17 | NA | chr2_25034310 |  |
|  | g47_FF(skin) | FarmCPU | 1.41E-25 | NA | chr4_16000421 |  |
|  | g48_FF(skin) | FarmCPU | 1.68E-25 | NA | chr4_16000421 |  |
|  | g49_FF(skin) | FarmCPU | 1.17E-09 | NA | chr7_25384148 |  |
| Fruit size (fruit lateral diameter) | g50_FS(FLaD) | FarmCPU | 3.38e−10 | 17.3 | PAV01_REGINA_50388238 | *P. avium* ‘Regina’ (Donkpegan *et al*., 2023) |
|  | g51_FS(FLaD) | FarmCPU | 3.23e−10 | 7.2 | PAV02_REGINA_4882204 |  |
|  | g52_FS(FLaD) | FarmCPU | 2.25e−07 | 3.1 | PAV06_REGINA_23191731 |  |
|  | g53_FS(FLaD) | FarmCPU | 2.98e−08 | 7.5 | PAV07_REGINA_18276189 |  |
|  | g54_FS(FLaD) | FarmCPU | 1.13e−07 | 6.9 | Pp01_34857341 | *P. persica* ‘PLov2-2n’ V2.0 (Donkpegan *et al*., 2023) |
|  | g55_FS(FLaD) | FarmCPU | 1.06e−10 | 8.6 | Pp02_9537634 |  |
|  | g56_FS(FLaD) | FarmCPU | 3.96e−07 | 12.6 | Pp04_20079115 |  |
|  | g57_FS(FLaD) | FarmCPU | 2.53E-13 | NA | chr2_29776725 | *P. avium* 'Tieton' V2.0 (Holušová *et al*., 2023) |
|  | g58_FS(FLaD) | FarmCPU | 6.68E-09 | NA | chr4_17924819 |  |
|  | g59_FS(FLaD) | FarmCPU | 1.34E-08 | NA | chr4_26059601 |  |
|  | g60_FS(FLaD) | FarmCPU | 1.81E-08 | NA | chr4_6228894 |  |
|  | g61_FS(FLaD) | FarmCPU | 2.70E-10 | NA | chr5_23342227 |  |
|  | g62_FS(FLaD) | FarmCPU | 2.10E-10 | NA | chr5_24274628 |  |
| Fruit size (fruit longitude diameter) | g63_FS (FLoD) | FarmCPU | 1.04E-18 | NA | chr1_43682223 |  |
|  | g64_FS (FLoD) | FarmCPU | 3.40E-10 | NA | chr2_29275642 |  |
|  | g65_FS (FLoD) | FarmCPU | 2.88E-16 | NA | chr2_29776624 |  |
|  | g66_FS (FLoD) | FarmCPU | 2.35E-09 | NA | chr4_5976608 |  |
|  | g67_FS (FLoD) | FarmCPU | 2.85E-10 | NA | chr5_11497974 |  |
|  | g68_FS (FLoD) | FarmCPU | 1.28E-11 | NA | chr6_34823266 |  |
|  | g69_FS (FLoD) | FarmCPU | 1.04E-10 | NA | chr8_27088406 |  |
|  | g70_FS (FLoD) | FarmCPU | 1.15E-15 | NA | chr8_28900983 |  |
| Fruit size ( fruit transverse diameter) | g71_FS (FTrD) | FarmCPU | 8.60e−08 | 15.0 | PAV01_REGINA_50388238 | *P. avium* ‘Regina’ (Donkpegan *et al*., 2023) |
|  | g72_FS (FTrD) | FarmCPU | 4.17e−11 | 8.0 | PAV07_REGINA_18276189 |  |
|  | g73_FS (FTrD) | FarmCPU | 4.01E-15 | NA | chr1_33914270 | *P. avium* 'Tieton' V2.0 (Holušová *et al*., 2023) |
|  | g74_FS (FTrD) | FarmCPU | 7.94E-32 | NA | chr2_31850964 |  |
|  | g75_FS (FTrD) | FarmCPU | 2.30E-08 | NA | chr2_7960492 |  |
|  | g76_FS (FTrD) | FarmCPU | 4.88E-09 | NA | chr3_18843170 |  |
|  | g77_FS (FTrD) | FarmCPU | 5.27E-09 | NA | chr4_16680250 |  |
|  | g78_FS (FTrD) | FarmCPU | 1.78E-13 | NA | chr5_24153701 |  |
|  | g79_FS (FTrD) | FarmCPU | 1.09E-09 | NA | chr8_14165946 |  |
|  | g80_FS (FTrD) | FarmCPU | 1.26E-08 | NA | chr8_19751357 |  |
| Fruit size ( fruit weight) | g81_FS (FW) | FarmCPU | 4.11e−13 | 5.1 | PAV01_REGINA_11561777 | *P. avium ‘*Regina’ (Donkpegan *et al., 2023)* |
|  | g82_FS (FW) | FarmCPU | 5.52e−10 | 18.8 | PAV01_REGINA_50388238 |  |
|  | g83_FS (FW) | FarmCPU | 7.96e−08 | 5.8 | PAV02_REGINA_4882204 |  |
|  | g84_FS (FW) | FarmCPU | 2.12e−07 | 17.2 | PAV04_REGINA_12024925 |  |
|  | g85_FS (FW) | FarmCPU | 8.87e−08 | 7.3 | Pp02_28325332 | *P. persica* ‘PLov2-2n’ V2.0 (Donkpegan et al., 2023) |
|  | g86_FS (FW) | FarmCPU | 1.20E-08 | NA | chr1_24330881 | *P. avium* 'Tieton' V2.0 (Holušová et al., 2023) |
|  | g87_FS (FW) | FarmCPU | 7.88E-09 | NA | chr1_31819328 |  |
|  | g88_FS (FW) | FarmCPU | 1.19E-09 | NA | chr1_33914270 |  |
|  | g89_FS (FW) | FarmCPU | 1.11E-23 | NA | chr2_29787028 |  |
|  | g90_FS (FW) | FarmCPU | 8.26E-09 | NA | chr4_5976608 |  |
|  | g91_FS (FW) | FarmCPU | 3.94E-11 | NA | chr6_16582783 |  |
| Fruit size (pit lateral diameter) | g92_FS (PLaD) | FarmCPU | 7.32e−15 | 27.3 | PAV_r1.0chr2_13099549 | *P. avium '*Satonishiki*'* (Donkpegan *et al.,* 2023) |
|  | g93_FS (PLaD) | FarmCPU | 3.69e−07 | 10.9 | PAV_r1.0chr3_12470834 |  |
|  | g94_FS (PLaD) | FarmCPU | 4.51e−10 | 14.8 | PAV_r1.0chr4_11934189 |  |
|  | g95_FS (PLaD) | FarmCPU | 1.35e−07 | 3.9 | PAV_r1.0chr4_17172922 |  |
|  | g96_FS (PLaD) | FarmCPU | 1.75e−07 | 4.4 | PAV08_REGINA_21099455 | *P. avium* ‘Regina’ (Donkpegan *et al*., 2023) |
|  | g97_FS (PLaD) | FarmCPU | 8.36e−08 | 6.3 | Pp05_986062 | *P. persica* ‘PLov2-2n’ V2.0 (Donkpegan *et al*., 2023) |
|  | g98_FS (PLaD) | FarmCPU | 2.10e−07 | 12.2 | Pp08_12886531 |  |
| Fruit size (pit transverse diameter) | g99_FS (PTrD) | FarmCPU | 6.79e−09 | 18.8 | PAV02_REGINA_2834950 | *P. avium* ‘Regina’ (Donkpegan *et al*., 2023) |
|  | g100_FS (PTrD) | FarmCPU | 1.32e−08 | 14.3 | PAV03_REGINA_1281929 |  |
|  | g101_FS (PTrD) | FarmCPU | 9.05e−09 | 11.1 | PAV04_REGINA_9562013 |  |
|  | g102_FS (PTrD) | FarmCPU | 6.49e−10 | 7.4 | PAV06_REGINA_3901121 |  |
|  | g103_FS (PTrD) | FarmCPU | 1.40e−08 | 13.3 | Pp03_4481012 | *P. persica* ‘PLov2-2n’ V2.0 (Donkpegan *et al*., 2023) |
| Penetration value | g104_PV | FarmCPU | 1.11E-09 | NA | chr1_14345045 | *P. avium* 'Tieton' V2.0 (Holušová *et al*., 2023) |
|  | g105_PV | FarmCPU | 2.51E-09 | NA | chr2_27724952 |  |
|  | g106_PV | FarmCPU | 1.05E-12 | NA | chr2_43495800 |  |
|  | g107_PV | FarmCPU | 6.29E-08 | NA | chr3_19735173 |  |
|  | g108_PV | FarmCPU | 3.36E-09 | NA | chr6_13575852 |  |
|  | g109_PV | FarmCPU | 1.14E-10 | NA | chr6_18346767 |  |
|  | g110_PV | FarmCPU | 1.20E-13 | NA | chr6_41860311 |  |
|  | g111_PV | FarmCPU | 6.92E-17 | NA | chr8_19215607 |  |
| Pit shape | g112_PS | FarmCPU | 5.52e−12 | 19.7 | Pp02_11962528 | *P. persica* ‘PLov2-2n’ V2.0 (Donkpegan *et al*., 2023) |
|  | g113_PS | FarmCPU | 8.12e−09 | 3.3 | Pp03_2049727 |  |
| **Phenology** | | | | | | |
| Maturity date | g114_MD | FarmCPU | 1.03E-09 | NA | chr1_21578907 | *P. avium* 'Tieton' V2.0 (Holušová *et al*., 2023) |
|  | g115_MD | FarmCPU | 7.21E-26 | NA | chr4_16353619 |  |
|  | g116_MD | FarmCPU | 2.57E-21 | NA | chr4_4523736 |  |
|  | g117_MD | FarmCPU | 1.48E-08 | NA | chr6_17030992 |  |
|  | g118_MD | FarmCPU | 6.18E-10 | NA | chr7_28655493 |  |
| Note: ^1^ Major GWAS loci are with strong evidence (*P*-value ＜ 0.05 and percentage of variance explained (PVE) ＞3); ^2^ the origin position of the GWAS loci were from different genomes; Example: pp01_ is from *Prunus persica* ‘PLov2-2n’ V2.0, PAV01_REGINA_ is from *Prunus avium* ‘Regina’ V1.0; PAV_r1.0chr1_ is from *Prunus avium* 'Satonishiki' V1.0; chr1_ is from *Prunus avium* 'Tieton' V2.0; -: blast failed; NA: not available. | | | | | | |

**Table S2. List of genetic architecture of main agronomic traits in the edible cherries**

| **Category**  **(in Fig.1)** | **Origin (species)** | **QTL/GWAS loci/Gene lable^1^** | **Chr (Tieton V2.0)** | **Position (Tieton V2.0) (Mb)** | **Function** | **Reference** |
| --- | --- | --- | --- | --- | --- | --- |
| Abiotic stress | *P. pseudocerasus* | ***CpCHS1^b^*** | 1 | 4.66 | enhance drought resistance | (Hou *et al*., 2022b) |
|  | *P. avium* | ***PaLectinL7*** | 2 | 40.05 | enhance salt tolerance; promoted lignin deposition | (Wu *et al*., 2023b) |
|  | *P. avium* | ***PaLectinL16*** | 4 | 14.96 | enhance resistance with abiotic (salt, drought) stresses | (Sun *et al*., 2022) |
|  | *P. cerasus* | ***PacCYP707A1*** | 5 | 17.69 | mediate drought tolerance | (Li *et al*., 2015) |
|  | *P. pseudocerasus* | ***CpARF7^b^*** | 7 | 26.34 | regulate drought and low phosphorus stress and root formation | (Hou *et al*., 2022a) |
| Biochemical | *P. avium* | q5_Neochlorogenicacid | 1 | 56.08~57.11 | Neochlorogenic acid | (Calle *et al*., 2021a) |
|  | *P. avium* | q7_p-CA | 1 | 56.08~56.6 | p-Coumaric acid | (Calle *et al*., 2021a) |
|  | *P. avium* | q8_p-CQA | 1 | 56.08~57.11 | p-Coumaroyl quinic acid | (Calle *et al*., 2021a) |
|  | *P. avium* | q3_CY3R | 3 | 14.55~24.88 | Cyanidin 3-O-rutinoside | (Calle *et al*., 2021a) |
|  | *P. avium* | q6_Neochlorogenicacid | 3 | 16.3~21.54 | Neochlorogenic acid | (Calle *et al*., 2021a) |
|  | *P. avium* | q1_CY3G | 3 | 18.26~27.83 | Cyanidin 3-O-glucoside | (Calle *et al*., 2021a) |
|  | *P. avium* | q4_CY3R | 3 | 21.54~24.12 | Cyanidin 3-O-rutinoside | (Calle *et al*., 2021a) |
|  | *P. avium* | q9_Pe3G | 3 | 21.54~24.12 | Peonidin 3-O-glucoside | (Calle *et al*., 2021a) |
|  | *P. avium* | q10_Pe3R | 3 | 21.62~22.03 | Peonidin 3-O-rutinoside | (Calle *et al*., 2021a) |
|  | *P. avium* | q2_CY3G | 3 | 24.1~24.75 | Cyanidin 3-O-glucoside | (Calle *et al*., 2021a) |
|  | *P. avium* | q11_Pe3R | 4 | 14.18~16.76 | Peonidin 3-O-rutinoside | (Calle *et al*., 2021a) |
|  | *P. avium* | ***PacLTP*** | 6 | 33.34 | elicit cherry allergy (for the Mediterranean population) | (Scheurer *et al*., 2001) |
|  | *P. cerasus* | ***PcSOT1^a^*** | 8 | 25.16 | regulate sorbitol and dry matter accumulation | (Gao *et al*., 2003) |
|  | *P. cerasus* | ***PcSOT2^a^*** | 8 | 25.18 | regulate sorbitol and dry matter accumulation | (Gao *et al*., 2003) |
| Fruit color | *P. avium* | g7_FC(flesh) | 1 | 41.00 | Fruit color (flesh) | (Holušová *et al*., 2023) |
|  | *P. avium* | g14_FC(skin) | 1 | 41.00 | Fruit color (skin) | (Holušová *et al*., 2023) |
|  | *P. avium* | g8_FC(flesh) | 2 | 3.07 | Fruit color (flesh) | (Holušová *et al*., 2023) |
|  | *P. avium* | ***PavGST1*** | 3 | 5.11 | promote fruits' anthocyanin accumulation | (Qi *et al*., 2022a) |
|  | *P. avium* | g17_FC(skin) | 3 | 6.27 | Fruit color (skin) | (Holušová *et al*., 2023) |
|  | *P. avium* | g15_FC(skin) | 3 | 19.32 | Fruit color (skin) | (Holušová *et al*., 2023) |
|  | *P. avium* | g9_FC(flesh) | 3 | 23.94 | Fruit color (flesh) | (Holušová *et al*., 2023) |
|  | *P. avium* | g16_FC(skin) | 3 | 23.94 | Fruit color (skin) | (Holušová *et al*., 2023) |
|  | *P. avium* | ***PavMYB10.1*** | 3 | 24.00 | determine fruit colors | (Jin *et al*., 2016) |
|  | *P. avium* | ***PacMYBA*** | 3 | 24.00 | promote anthocyanin accumulation in red-colored fruit | (Shen *et al*., 2014) |
|  | *P. avium* | g10_FC(flesh) | 3 | 24.54 | Fruit color (flesh) | (Holušová *et al*., 2023) |
|  | *P. avium* | g11_FC(flesh) | 3 | 25.10 | Fruit color (flesh) | (Holušová *et al*., 2023) |
|  | *P. avium* | ***PavBBX6*** | 3 | 30.30 | promote anthocyanin accumulation | (Wang *et al*., 2023b) |
|  | *P. avium* | q15_FC(skin) | 3 | 12.35~29.98 | Fruit color (skin) | (Calle *et al*., 2021a) |
|  | *P. avium* | q12_FC(flesh) | 3 | 13.51~31.5 | Fruit color (flesh) | (Calle *et al*., 2021a) |
|  | *P. avium* | q13_FC(flesh) | 3 | 21.54~33.79 | Fruit color (flesh) | (Calle *et al*., 2021a) |
|  | *P. avium* | q16_FC(skin) | 3 | 21.98~32.54 | Fruit color (skin) | (Calle *et al*., 2021a) |
|  | *P. avium* | q17_FC(skin)/q14_FC(flesh)/q18_FC(skin) | 3 | 23.54~34.49 | Fruit color (skin)/(flesh) | (Sooriyapathirana *et al*., 2010) |
|  | *P. avium* | ***PavNCED1*** | 4 | 12.22 | promotes anthocyanin biosynthesis; promote ABA biosynthesis | (Shen *et al*., 2014; Zhai *et al*., 2022) |
|  | *P. avium* | ***PavBBX9*** | 4 | 12.71 | promote anthocyanin accumulation | (Wang *et al*., 2023b) |
|  | *P. avium* | g18_FC(skin) | 4 | 15.75 | Fruit color (skin) | (Holušová *et al*., 2023) |
|  | *P. avium* | ***PacCOP1*** | 5 | 19.95 | negatively regulate anthocyanin biosynthesis | (Liang *et al*., 2020) |
|  | *P. avium* | g12_FC(flesh) | 6 | 24.85 | Fruit color (flesh) | (Holušová *et al*., 2023) |
|  | *P. avium* | g13_FC(flesh) | 7 | 18.66 | Fruit color (flesh) | (Holušová *et al*., 2023) |
| Fruit cracking | *P. avium* | g23_FCr(PE) | 1 | 5.02 | Fruit cracking (pistillar end) | (Donkpegan *et al*., 2023) |
|  | *P. avium* | g19_FCr | 1 | 21.35 | Fruit cracking | (Crump *et al*., 2022) |
|  | *P. avium* | g38_FCr(SE) | 1 | 23.70 | Fruit cracking (stem end) | (Donkpegan *et al*., 2023) |
|  | *P. avium* | g32_FCr(SE) | 1 | 41.23 | Fruit cracking (stem end) | (Donkpegan *et al*., 2023) |
|  | *P. avium* | q19_FCr | 1 | 18.77~33.75 | Fruit cracking | (Crump *et al*., 2022) |
|  | *P. avium* | ***PaPIP1-4*** | 2 | 40.52 | prevent cracking by pre-harvest calcium treatments | (Breia *et al*., 2020) |
|  | *P. avium* | q21_FCr(FS) | 2 | 12.13~25.37 | Fruit cracking (fruit side) | (Quero-García *et al*., 2021) |
|  | *P. avium* | q22_FCr(FS) | 2 | 27.39~44.39 | Fruit cracking (fruit side) | (Quero-García *et al*., 2021) |
|  | *P. avium* | g20_FCr | 3 | 22.40 | Fruit cracking | (Crump *et al*., 2022) |
|  | *P. avium* | g33_FCr(SE) | 4 | 4.75 | Fruit cracking (stem end) | (Donkpegan *et al*., 2023) |
|  | *P. avium* | g34_FCr(SE) | 4 | 4.75 | Fruit cracking (stem end) | (Donkpegan *et al*., 2023) |
|  | *P. avium* | g24_FCr(PE) | 4 | 6.82 | Fruit cracking (pistillar end) | (Donkpegan *et al*., 2023) |
|  | *P. avium* | g28_FCr(PE) | 4 | 18.81 | Fruit cracking (pistillar end) | (Donkpegan *et al*., 2023) |
|  | *P. avium* | g29_FCr(PE) | 4 | 18.81 | Fruit cracking (pistillar end) | (Donkpegan *et al*., 2023) |
|  | *P. avium* | q25_FCr(PE) | 4 | 5.68~9.09 | Fruit cracking (pistillar end) | (Quero-García *et al*., 2021) |
|  | *P. avium* | q26_FCr(PE) | 4 | 26.8~27.62 | Fruit cracking (pistillar end) | (Quero-García *et al*., 2021) |
|  | *P. avium* | g30_FCr(PE) | 5 | 18.06 | Fruit cracking (pistillar end) | (Donkpegan *et al*., 2023) |
|  | *P. avium* | g21_FCr | 5 | 31.86 | Fruit cracking | (Crump *et al*., 2022) |
|  | *P. avium* | q27_FCr(PE) | 5 | 20.34~23.58 | Fruit cracking (pistillar end) | (Quero-García *et al*., 2021) |
|  | *P. avium* | q28_FCr(PE) | 5 | 21.61~23.98 | Fruit cracking (pistillar end) | (Quero-García *et al*., 2021) |
|  | *P. avium* | q29_FCr(PE) | 5 | 28.01~29.17 | Fruit cracking (pistillar end) | (Quero-García *et al*., 2021) |
|  | *P. avium* | q30_FCr(PE) | 5 | 30.22~32.52 | Fruit cracking (pistillar end) | (Quero-García *et al*., 2021) |
|  | *P. avium* | q20_FCr | 5 | 30.6~33.28 | Fruit cracking | (Crump *et al*., 2022) |
|  | *P. avium* | g25_FCr(PE) | 6 | 24.69 | Fruit cracking (pistillar end) | (Donkpegan *et al*., 2023) |
|  | *P. avium* | g26_FCr(PE) | 6 | 35.00 | Fruit cracking (pistillar end) | (Donkpegan *et al*., 2023) |
|  | *P. avium* | q31_FCr(SE) | 6 | 25.48~27.56 | Fruit cracking (stem end) | (Quero-García *et al*., 2021) |
|  | *P. avium* | q32_FCr(SE) | 6 | 33.65~35.16 | Fruit cracking (stem end) | (Quero-García *et al*., 2021) |
|  | *P. avium* | g31_FCr(PE) | 7 | 14.08 | Fruit cracking (pistillar end) | (Donkpegan *et al*., 2023) |
|  | *P. avium* | g36_FCr(SE) | 7 | 22.57 | Fruit cracking (stem end) | (Donkpegan *et al*., 2023) |
|  | *P. avium* | g27_FCr(PE) | 7 | 25.47 | Fruit cracking (pistillar end) | (Donkpegan *et al*., 2023) |
|  | *P. avium* | g37_FCr(SE) | 7 | 27.86 | Fruit cracking (stem end) | (Donkpegan *et al*., 2023) |
|  | *P. avium* | g35_FCr(SE) | 7 | 28.51 | Fruit cracking (stem end) | (Donkpegan *et al*., 2023) |
|  | *P. avium* | q23_FCr(FS) | 7 | 0~7.56 | Fruit cracking (fruit side) | (Quero-García *et al*., 2021) |
|  | *P. avium* | q24_FCr(FS) | 7 | 28.7~29.55 | Fruit cracking (fruit side) | (Quero-García *et al*., 2021) |
|  | *P. avium* | g41_FCr(SE) | 8 | 15.49 | Fruit cracking (stem end) | (Donkpegan *et al*., 2023) |
|  | *P. avium* | g22_FCr | 8 | 31.82 | Fruit cracking | (Crump *et al*., 2022) |
| Fruit firmness | *P. avium* | ***PpsGalAK-like^b^*** | 1 | 9.16 | increase protopectin content | (Jiu *et al*., 2024) |
|  | *P. avium* | g44_FF(skin) | 1 | 31.86 | Fruit firmness (skin) | (Holušová*et al*., 2023) |
|  | *P. avium* | ***PavDof2*** | 1 | 44.39 | delay softening | (Zhai *et al*., 2022) |
|  | *P. avium* | g45_FF(skin) | 1 | 58.90 | Fruit firmness (skin) | (Holušová *et al*., 2023) |
|  | *P. avium* | q33_FF | 1 | 15.94~34.9 | Fruit firmness | (Crump *et al*., 2022) |
|  | *P. avium* | q34_FF | 1 | 18.04~26.71 | Fruit firmness | (Calle *et al*., 2020a) |
|  | *P. avium* | q35_FF | 1 | 20.73~32.65 | Fruit firmness | (Calle *et al*., 2020a) |
|  | *P. avium* | g46_FF(skin) | 2 | 25.03 | Fruit firmness (skin) | (Holušová*et al*., 2023) |
|  | *P. avium* | ***PavDof6*** | 2 | 44.31 | precocious, promote softening | (Zhai *et al*., 2022) |
|  | *P. avium* | g43_FF | 3 | 9.22 | Fruit firmness | (Crump *et al*., 2022) |
|  | *P. avium* | ***PaMADS7*** | 3 | 32.12 | positively regulate fruit ripening and softening | (Zhai *et al*., 2022; Qi *et al*., 2022b) |
|  | *P. avium* | q36_FF | 3 | 24.91~29.41 | Fruit firmness | (Crump *et al*., 2022) |
|  | *P. avium* | ***PavARF8*** | 4 | 3.43 | delayed softening | (Zhai *et al*., 2022) |
|  | *P. avium* | ***PavPG38*** | 4 | 8.59 | reduce fruit firmness | (Zhai *et al*., 2021) |
|  | *P. avium* | g47_FF(skin) | 4 | 16.00 | Fruit firmness (skin) | (Holušová *et al*., 2023) |
|  | *P. avium* | g48_FF(skin) | 4 | 16.00 | Fruit firmness (skin) | (Holušová *et al*., 2023) |
|  | *P. avium* | ***PavNAC56*** | 4 | 16.01 | promote ripening and softening | (Qi *et al*., 2022a) |
|  | *P. avium* | q37_FF | 4 | 14.18~16.76 | Fruit firmness | (Calle & Wünsch, 2020) |
|  | *P. avium* | q38_FF/q39_FF | 4 | 14.92~16.09 | Fruit firmness | (Cai *et al*., 2019) |
|  | *P. avium* | ***PavDof15*** | 5 | 33.18 | delayed softening | (Zhai *et al*., 2022) |
|  | *P. avium* | q40_FF | 5 | 21.34~33.2 | Fruit firmness | (Campoy *et al*., 2015) |
|  | *P. avium* | q41_FF | 5 | 33.2~33.2 | Fruit firmness | (Campoy *et al*., 2015) |
|  | *P. avium* | ***PavXTH14*** | 6 | 10.78 | reduce fruit firmness | (Zhai *et al*., 2021) |
|  | *P. avium* | ***PpsStv1^b^*** | 6 | 13.13 | increase protopectin content | (Jiu *et al*., 2024) |
|  | *P. avium* | g49_FF(skin) | 7 | 25.38 | Fruit firmness (skin) | (Holušová *et al*., 2023) |
|  | *P. avium* | ***PavXTH15*** | 8 | 33.07 | reduce fruit firmness | (Zhai *et al*., 2021) |
| Fruit size | *P. avium* | g81_FS(FW) | 1 | 15.88 | Fruit size ( fruit weight) | (Donkpegan *et al*., 2023) |
|  | *P. avium* | g86_FS(FW) | 1 | 24.33 | Fruit size ( fruit weight) | (Holušová *et al*., 2023) |
|  | *P. avium* | g87_FS(FW) | 1 | 31.82 | Fruit size ( fruit weight) | (Holušová *et al*., 2023) |
|  | *P. avium* | g73_FS(FTrD) | 1 | 33.91 | Fruit size ( fruit transverse diameter) | (Holušová *et al*., 2023) |
|  | *P. avium* | g88_FS(FW) | 1 | 33.91 | Fruit size ( fruit weight) | (Holušová *et al*., 2023) |
|  | *P. avium* | g63_FS(FLoD) | 1 | 43.68 | Fruit size (fruit longitude diameter) | (Holušová *et al*., 2023) |
|  | *P. avium* | g54_FS(FLaD) | 1 | 44.51 | Fruit size (fruit lateral diameter) | (Donkpegan *et al*., 2023) |
|  | *P. avium* | g50_FS(FLaD) | 1 | 55.57 | Fruit size (fruit lateral diameter) | (Donkpegan *et al*., 2023) |
|  | *P. avium* | g71_FS(FTrD) | 1 | 55.57 | Fruit size ( fruit transverse diameter) | (Donkpegan *et al*., 2023) |
|  | *P. avium* | g82_FS(FW) | 1 | 55.57 | Fruit size ( fruit weight) | (Donkpegan *et al*., 2023) |
|  | *P. avium* | q50_FS(FW) | 1 | 24.28~37.56 | Fruit size (fruit weight) | (Rosyara *et al*., 2013) |
|  | *P. avium* | q49_FS(FW) | 1 | 37.56~41.26 | Fruit size (fruit weight) | (Calle *et al*., 2020a) |
|  | *P. avium* | g75_FS(FTrD) | 2 | 7.96 | Fruit size ( fruit transverse diameter) | (Holušová *et al*., 2023) |
|  | *P. avium* | ***PavAGL15*** | 2 | 9.12 | increase fruit size | (Dong *et al*., 2022) |
|  | *P. avium* | g99_FS(PTrD) | 2 | 9.34 | Fruit size (pit transverse diameter) | (Donkpegan *et al*., 2023) |
|  | *P. avium* | g51_FS(FLaD) | 2 | 11.41 | Fruit size (fruit lateral diameter) | (Donkpegan *et al*., 2023) |
|  | *P. avium* | g83_FS(FW) | 2 | 11.41 | Fruit size ( fruit weight) | (Donkpegan *et al*., 2023) |
|  | *P. avium* | g64_FS(FLoD) | 2 | 29.28 | Fruit size (fruit longitude diameter) | (Holušová *et al*, 2023) |
|  | *P. avium* | g65_FS(FLoD) | 2 | 29.78 | Fruit size (fruit longitude diameter) | (Holušová *et al*., 2023) |
|  | *P. avium* | g58_FS(FLaD) | 2 | 29.78 | Fruit size (fruit lateral diameter) | (Holušová *et al*., 2023) |
|  | *P. avium* | g89_FS(FW) | 2 | 29.79 | Fruit size ( fruit weight) | (Holušová *et al*., 2023) |
|  | *P. avium* | g74_FS(FTrD) | 2 | 31.85 | Fruit size ( fruit transverse diameter) | (Holušová *et al*., 2023) |
|  | *P. avium* | ***PaCYP78A9*** | 2 | 40.11 | increase fruit size | (Qi et al., 2017) |
|  | *P. avium* | g85_FS(FW) | 2 | 42.90 | Fruit size ( fruit weight) | (Donkpegan *et al*., 2023) |
|  | *P. avium* | q51_FS(FW) | 2 | 8.11~30.61 | Fruit size (fruit weight) | (Rosyara *et al*., 2013) |
|  | *P. avium* | q43&44_FLoD/q46&47_FTrD/q56_FW/q57_FW/q61_MCN/q62&63_MTrD/q64&65_MLoD | 2 | 28.44~33.69 | Fruit size | (Zhang *et al*., 2010) |
|  | *P. avium* | q55_FS(FW) | 2 | 29.77~39.17 | Fruit size (fruit weight) | (Campoy *et al*., 2015) |
|  | *P. avium* | q54_FS(FW) | 2 | 30.35~33.28 | Fruit size (fruit weight) | (Campoy *et al*., 2015) |
|  | *P. avium* | q52_FS(FW) | 2 | 33.28~33.57 | Fruit size (fruit weight) | (Rosyara *et al*., 2013) |
|  | *P. avium* | q42_FS | 2 | 37.91~41.29 | Fruit size | (Calle & Wünsch, 2020) |
|  | *P. avium* | g100_FS(PTrD) | 3 | 5.33 | Fruit size (pit transverse diameter) | (Donkpegan *et al*., 2023) |
|  | *P. avium* | g103_FS(PTrD) | 3 | 9.20 | Fruit size (pit transverse diameter) | (Donkpegan *et al*., 2023) |
|  | *P. avium* | g76_FS(FTrD) | 3 | 18.84 | Fruit size ( fruit transverse diameter) | (Holušová *et al*., 2023) |
|  | *P. avium* | g93_FS(PLaD) | 3 | 24.54 | Fruit size (pit lateral diameter) | (Donkpegan *et al*., 2023) |
|  | *P. avium* | ***PavRAV2*** | 3 | 31.27 | decrease fruit size (mesocarp cell expansion) | (Qi *et al*., 2023) |
|  | *P. avium* | q57_FS(FW) | 3 | 20.83~34.49 | Fruit size (fruit weight) | (Zhang *et al*., 2010) |
|  | *P. avium* | g66_FS(FLoD) | 4 | 5.98 | Fruit size (fruit longitude diameter) | (Holušová *et al*., 2023) |
|  | *P. avium* | g90_FS(FW) | 4 | 5.98 | Fruit size ( fruit weight) | (Holušová *et al*., 2023) |
|  | *P. avium* | g60_FS(FLaD) | 4 | 6.23 | Fruit size (fruit lateral diameter) | (Holušová *et al.*, 2023) |
|  | *P. avium* | g101_FS(PTrD) | 4 | 10.87 | Fruit size (pit transverse diameter) | (Donkpegan et al., 2023) |
|  | *P. avium* | g94_FS(PLaD) | 4 | 13.07 | Fruit size (pit lateral diameter) | (Donkpegan *et al.*, 2023) |
|  | *P. avium* | g84_FS(FW) | 4 | 13.21 | Fruit size ( fruit weight) | (Donkpegan *et al*., 2023) |
|  | *P. avium* | g77_FS(FTrD) | 4 | 16.68 | Fruit size ( fruit transverse diameter) | (Holušová *et al.,* 2023) |
|  | *P. avium* | g58_FS(FLaD) | 4 | 17.92 | Fruit size (fruit lateral diameter) | (Holušová *et al*., 2023) |
|  | *P. avium* | g59_FS(FLaD) | 4 | 26.06 | Fruit size (fruit lateral diameter) | (Holušová *et al.*, 2023) |
|  | *P. avium* | g67_FS(FLoD) | 5 | 11.50 | Fruit size (fruit longitude diameter) | (Holušová *et al*., 2023) |
|  | *P. avium* | g97_FS(PLaD) | 5 | 18.21 | Fruit size (pit lateral diameter) | (Donkpegan *et al*., 2023) |
|  | *P. avium* | g61_FS(FLaD) | 5 | 23.34 | Fruit size (fruit lateral diameter) | (Holušová *et al*., 2023) |
|  | *P. avium* | g78_FS(FTrD) | 5 | 24.15 | Fruit size ( fruit transverse diameter) | (Holušová *et al*., 2023) |
|  | *P. avium* | g62_FS(FLaD) | 5 | 24.27 | Fruit size (fruit lateral diameter) | (Holušová *et al*., 2023) |
|  | *P. avium* | ***PaCYP78A6*** | 5 | 28.23 | increase fruit size | (Qi *et al*., 2019) |
|  | *P. avium* | ***PavKLUH*** | 5 | 33.03 | increase fruit size (mesocarp cell expansion) | (Qi *et al*., 2023) |
|  | *P. avium* | g102_FS(PTrD) | 6 | 6.50 | Fruit size (pit transverse diameter) | (Donkpegan *et al.*, 2023) |
|  | *P. avium* | g91_FS(FW) | 6 | 16.58 | Fruit size ( fruit weight) | (Holušová *et al*., 2023) |
|  | *P. avium* | g52_FS(FLaD) | 6 | 25.65 | Fruit size (fruit lateral diameter) | (Donkpegan *et al.*, 2023) |
|  | *P. avium* | g68_FS(FLoD) | 6 | 34.82 | Fruit size (fruit longitude diameter) | (Holušová *et al*., 2023) |
|  | *P. avium* | q60_FS(FW)/q45_FS(FLoD)/q48_FS(FTrD)/q66_FS(PTrD)/q67_FS(PLoD) | 6 | 28.03~29.52 | Fruit size | (Zhang *et al.*, 2010) |
|  | *P. avium* | g53_FS(FLaD) | 7 | 19.18 | Fruit size (fruit lateral diameter) | (Donkpegan *et al*., 2023) |
|  | *P. avium* | g72_FS(FTrD) | 7 | 19.18 | Fruit size ( fruit transverse diameter) | (Donkpegan *et al*., 2023) |
|  | *P. avium* | g79_FS(FTrD) | 8 | 14.17 | Fruit size ( fruit transverse diameter) | (Holušová *et al*., 2023) |
|  | *P. avium* | g80_FS(FTrD) | 8 | 19.75 | Fruit size ( fruit transverse diameter) | (Holušová *et al.*, 2023) |
|  | *P. avium* | g98_FS(PLaD) | 8 | 24.72 | Fruit size (pit lateral diameter) | (Donkpegan *et al.*, 2023) |
|  | *P. avium* | g69_FS(FLoD) | 8 | 27.09 | Fruit size (fruit longitude diameter) | (Holušová *et al.*, 2023) |
|  | *P. avium* | g70_FS(FLoD) | 8 | 28.90 | Fruit size (fruit longitude diameter) | (Holušová *et al*., 2023) |
|  | *P. avium* | g96_FS(PLaD) | 8 | 31.39 | Fruit size (pit lateral diameter) | (Donkpegan *et al*., 2023) |
| Fruit other traits | *P. avium* | g1_FB | 1 | 6.10 | Fruit bruiseness | (Holušová *et al*., 2023) |
|  | *P. avium* | g104_PV | 1 | 14.35 | Penetration value | (Holušová *et al*., 2023) |
|  | *P. avium* | g2_FB | 1 | 61.83 | Fruit bruiseness | (Holušová *et al*., 2023) |
|  | *P. avium* | g112_PS | 2 | 23.11 | Pit shape | (Donkpegan *et al*., 2023) |
|  | *P. avium* | g105_PV | 2 | 27.72 | Penetration value | (Holušová *et al*., 2023) |
|  | *P. avium* | g106_PV | 2 | 43.50 | Penetration value | (Holušová *et al*., 2023) |
|  | *P. avium* | g113_PS | 3 | 6.26 | Pit shape | (Donkpegan *et al*., 2023) |
|  | *P. avium* | g107_PV | 3 | 19.74 | Penetration value | (Holušová *et al*., 2023) |
|  | *P. avium* | g3_FB | 3 | 26.05 | Fruit bruiseness | (Holušová *et al*., 2023) |
|  | *P. avium* | g4_FB | 4 | 16.23 | Fruit bruiseness | (Holušová *et al*., 2023) |
|  | *P. avium* | q68_SSC | 4 | 14.18~17.92 | Soluble solid content | (Calle & Wünsch, 2020) |
|  | *P. avium* | g108_PV | 6 | 13.58 | Penetration value | (Holušová *et al*., 2023) |
|  | *P. avium* | g109_PV | 6 | 18.35 | Penetration value | (Holušová *et al.*, 2023) |
|  | *P. avium* | g5_FB | 6 | 34.67 | Fruit bruiseness | (Holušová *et al*., 2023) |
|  | *P. avium* | g110_PV | 6 | 41.86 | Penetration value | (Holušová *et al*., 2023) |
|  | *P. avium* | q69_TA | 6 | 33.2~36.86 | Total acidity | (Calle & Wünsch, 2020) |
|  | *P. avium* | g6_FB | 7 | 25.38 | Fruit bruiseness | (Holušová *et al*., 2023) |
|  | *P. avium* | g111_PV | 8 | 19.22 | Penetration value | (Holušová *et al*., 2023) |
| Phenology | *P. avium* | ***PavGA2ox-2L*** | 1 | 13.69 | delays flowering time, promote dwarf dense planting and inhibits seed germination | (Liu *et al*., 2022b) |
|  | *P. avium* | g114_MD | 1 | 21.58 | Maturity date | (Holušová *et al*., 2023) |
|  | *P. avium* | q71_FD | 1 | 18.22~20.67 | Flowering date | (Branchereau *et al*., 2022) |
|  | *P. cerasus* | **q72_FD*^a^*** | 1 | 37.39~42.76 | Flowering date | (Cai et al., 2018) |
|  | *P. avium* | q73_FD | 1 | 40.21~54.31 | Flowering date | (Branchereau *et al*., 2022) |
|  | *P. avium* | q85_FD(beginning) | 1 | 52.13~56.18 | Flowering date (beginning) | (Branchereau *et al*., 2022) |
|  | *P. avium* | q74_FD | 1 | 54.26~60.06 | Flowering date | (Dirlewanger *et al*., 2012) |
|  | *P. avium* | q75_FD | 1 | 54.48~54.79 | Flowering date | (Calle *et al*., 2020b) |
|  | *P. cerasus* | q76_FD^a^ | 2 | 27.91~33.78 | Flowering date | (Cai *et al*., 2018) |
|  | *P. avium* | q77_FD | 2 | 33.75~35.77 | Flowering date | (Calle *et al*., 2020b) |
|  | *P. avium* | q89_MD | 2 | 39.17~41.29 | Maturity date | (Calle & Wünsch, 2020) |
|  | *P. avium* | ***PavSEP*** | 3 | 32.12 | shorten vegetative phase and promote early flowering | (Wang *et al*., 2021a) |
|  | *P. avium* | g116_MD | 4 | 4.52 | Maturity date | (Holušová*et al*., 2023) |
|  | *P. avium* | ***PavNCED5*** | 4 | 5.42 | enhanced seed and flower bud dormancy | (Wang *et al*., 2023d) |
|  | *P. avium* | g115_MD | 4 | 16.35 | Maturity date | (Holušová *et al*., 2023) |
|  | *P. avium* | q86_FD(beginning) | 4 | 0~0.8 | Flowering date (beginning) | (Branchereau *et al*., 2022) |
|  | *P. avium* | q70_CR | 4 | 10.3~12.39 | Chilling requirement | (Castede *et al*., 2014) |
|  | *P. avium* | q78_FD | 4 | 10.3~11.27 | Flowering date | (Castede *et al*., 2014) |
|  | *P. avium* | q79_FD | 4 | 10.39~10.59 | Flowering date | (Branchereau *et al*., 2022) |
|  | *P. cerasus* | q80_FD***^a^*** | 4 | 10.55~17.35 | Flowering date | (Cai *et al*., 2018) |
|  | *P. avium* | q81_FD | 4 | 12.7~14.04 | Flowering date | (Castede *et al*., 2014) |
|  | *P. avium* | q82_FD | 4 | 14.04~14.08 | Flowering date | (Dirlewanger *et al*., 2012) |
|  | *P. avium* | q88_FDP | 4 | 15.14~16.33 | Fruit development period | (Calle & Wünsch, 2020) |
|  | *P. avium* | q90_MD | 4 | 15.14~16.33 | Maturity date | (Calle & Wünsch, 2020) |
|  | *P. avium* | ***PavCIG1*** | 5 | 25.25 | delayed flowering | (Wang *et al*., 2021b) |
|  | *P. avium* | ***PavCIG2*** | 5 | 25.26 | repress flowering and maintain the dormancy status | (Wang *et al*., 2021b) |
|  | *P. avium* | ***PavFUL*** | 5 | 33.10 | led to early flowering and multi-silique formation | (Wang *et al*., 2022c) |
|  | *P. avium* | ***PavTCP17*** | 5 | 33.53 | positively regulate flower bud dormancy | (Wen *et al*., 2023) |
|  | *P. cerasus* | **q83_FD*^a^*** | 5 | 23.52~25.73 | Flowering date | (Cai *et al*., 2018) |
|  | *P. avium* | g117_MD | 6 | 17.03 | Maturity date | (Holušová *et al*., 2023) |
|  | *P. avium* | ***PavSVP*** | 6 | 26.16 | maintain suppression phase of flowering | (Wang *et al*., 2021a) |
|  | *P. avium* | ***PavFT*** | 6 | 37.56 | promote flowering | (Yarur *et al*., 2016) |
|  | *P. avium* | q87_FD(beginning) | 6 | 8.6~11.58 | Flowering date (beginning) | (Branchereau *et al*., 2022) |
|  | *P. avium* | ***PacCYP707A2*** | 7 | 22.51 | negatively regulate cherry fruit ripening | (Li *et al*., 2015) |
|  | *P. avium* | g118_MD | 7 | 28.66 | Maturity date | (Holušová*et al*., 2023) |
|  | *P. avium* | q84_FD | 7 | 22.98~28.65 | Flowering date | (Branchereau *et al*., 2022) |
| Physiology | *P. avium* | ***PavDAM1*** | 1 | 54.64 | result in abnormal flower and seed development | (Wang *et al*., 2020c) |
|  | *P. avium* | ***PavDAM5*** | 1 | 54.70 | result in abnormal flower and seed development | (Wang *et al*., 2020c) |
|  | *P. avium* | ***PavSOC1*** | 2 | 33.89 | result in abnormal flower and seed development | (Wang *et al*., 2020c) |
|  | *P. avium* | ***PaNRT2.1*** | 6 | 10.53 | regulate nitrate signalling pathways | (Wu *et al*., 2023a) |
|  | *P. avium* | ***PaLAX1*** | 6 | 31.72 | promote cell uptake of auxin | (Hoyerová *et al*., 2008) |
|  | *P. avium* | ***S4-SLFL2*** | 6 | 34.99 | mediate the ubiquitination and degradation of S-RNase | (Li *et al*., 2020) |
|  | *P. avium* | q91_TD | 7 | 2.62~7.83 | Trunk diameter | (Wang *et al*., 2015) |
|  | *P. avium* | q92_TD | 7 | 4.88~17.27 | Trunk diameter | (Wang *et al*., 2015) |
|  | *P. avium* | q93_TD | 7 | 5.89~11.06 | Trunk diameter | (Wang *et al*., 2015) |
|  | *P. avium* | q94_TD | 8 | 22.37~23.11 | Trunk diameter | (Wang *et al*., 2015) |

Note: ^1^ Label with ^a^ is detected from sour cherry, label with ^b^ is detected from Chinese cherry; others are detected from sweet cherry, consistent with Fig.1; and functional genes are indicated in bold italics.
